# A Simple Add-and-Display Method for Immobilisation of Cancer Drug on His-tagged Virus-like Nanoparticles for Controlled Drug Delivery

**DOI:** 10.1038/s41598-017-05525-4

**Published:** 2017-07-13

**Authors:** Roya Biabanikhankahdani, Saadi Bayat, Kok Lian Ho, Noorjahan Banu Mohamed Alitheen, Wen Siang Tan

**Affiliations:** 1 Department of Microbiology, Faculty of Biotechnology and Biomolecular Sciences, Universiti Putra Malaysia, 43400 UPM Serdang, Selangor Malaysia; 2 Department of Chemistry, Faculty of Science, Universiti Putra Malaysia, 43400 UPM Serdang, Selangor Malaysia; 3 Department of Pathology, Faculty of Medicine and Health Sciences, Universiti Putra Malaysia, 43400 UPM Serdang, Selangor Malaysia; 4 Department of Cell and Molecular Biology, Faculty of Biotechnology and Biomolecular Sciences, Universiti Putra Malaysia, 43400 UPM Serdang, Selangor Malaysia; 5 Institute of Bioscience, Universiti Putra Malaysia, 43400 UPM Serdang, Selangor Malaysia

## Abstract

pH-responsive virus-like nanoparticles (VLNPs) hold promising potential as drug delivery systems for cancer therapy. In the present study, hepatitis B virus (HBV) VLNPs harbouring His-tags were used to display doxorubicin (DOX) via nitrilotriacetic acid (NTA) conjugation. The His-tags served as pH-responsive nanojoints which released DOX from VLNPs in a controlled manner. The His-tagged VLNPs conjugated non-covalently with NTA-DOX, and cross-linked with folic acid (FA) were able to specifically target and deliver the DOX into ovarian cancer cells via folate receptor (FR)-mediated endocytosis. The cytotoxicity and cellular uptake results revealed that the His-tagged VLNPs significantly increased the accumulation of DOX in the ovarian cancer cells and enhanced the uptake of DOX, which improved anti-tumour effects. This study demonstrated that NTA-DOX can be easily displayed on His-tagged VLNPs by a simple Add-and-Display step with high coupling efficiency and the drug was only released at low pH in a controlled manner. This approach facilitates specific attachment of any drug molecule on His-tagged VLNPs at the very mild conditions without changing the biological structure and native conformation of the VLNPs.

## Introduction

Novel drug delivery systems which can release their payload in response to stimuli have received much attention lately. A variety of smart nanomaterials with a triggered smart mechanism responding to specific stimuli have been developed. These smart nanocarriers could release drugs in response to either physical stimuli (temperature, ultrasonic, and electrochemical), chemical stimuli (pH, redox, and ionic), or biological stimuli (glucose, enzymes, and inflammation). Among these different types of stimuli, pH-responsive nanoparticles appear to be the most attractive candidates^1–6^. These smart delivery systems are stable in a physiological environment (blood, pH = 7.4) but release the drug in an acidic environment (lysosome, pH = 5.0), resulting in an enhanced anti-tumour efficacy, and lower drug side effects^7, 8^.

**Figure 1:**
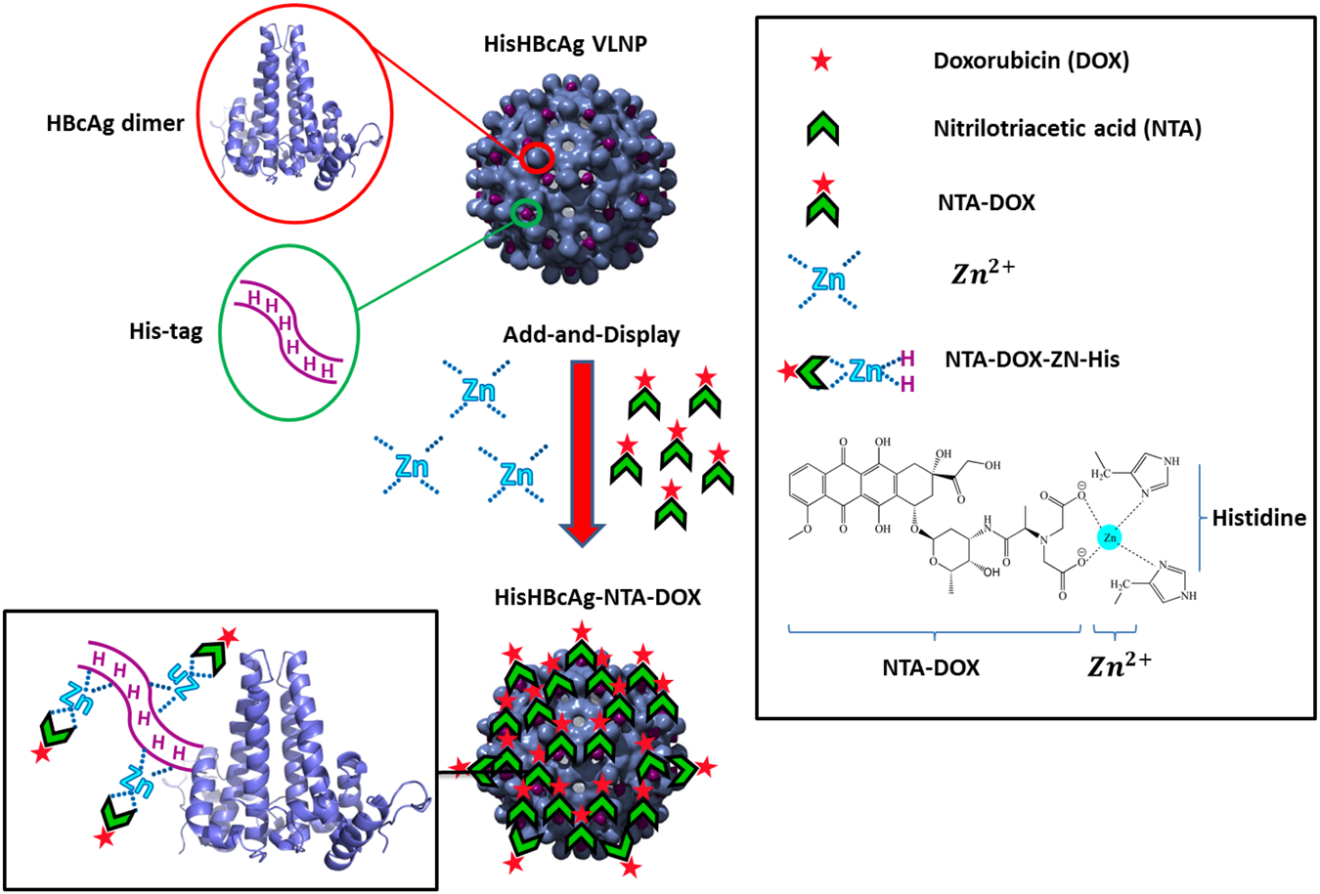
Schematic representation of the Add-and-Display method for immobilisation of doxorubicin non-covalently on His-tagged VLNPs. The HisHBcAg VLNP is made up of many copies of HBcAg dimers (blue). The His-tag fused at the N-terminal end of the HBcAg monomer forms trimeric spikes (magenta) and are exposed on the surface of the HisHBcAg VLNP. NTA-DOX is synthesised from nitrilotriacetic acid and doxorubicin hydrochloride. In the presence of Zn^2+^, the NTA-DOX interacts with histidine residues and is displayed on the surface of the VLNP.

Recently, we established pH-responsive virus-like nanoparticles (VLNPs) based on hepatitis B core antigen (HBcAg)^9^. Doxorubicin (DOX) and polyacrylic acid (PAA) were loaded together inside the VLNPs, while folic acid (FA) was conjugated on the surface of the particles via the nanoglue^9^. In the present study, we introduce a novel approach to display an anti-cancer drug, DOX, on the HBcAg VLNPs by exploiting the hexahistidine-tag (His-tag) exposed on the surface of the particles (Fig. 1). This approach does not involve a time-consuming drug packaging step and it allows any drug that binds to the His-tags to be displayed easily on the surface of VLNPs by a simple Add-and-Display step.

Yap *et al*.^10^ demonstrated that the HBcAg fused with a His-tag at its N-terminal end (HisHBcAg) self-assembled into VLNPs, which facilitated large-scale purification of the VLNPs by an immobilised metal affinity chromatography (IMAC) for diagnostic purposes. Cryo-electron microscopy and three-dimensional image reconstruction revealed that the His-tag formed a trimeric spike exposed on the surface of the VLNPs^11^. In the present study, this His-tag was exploited to bind DOX non-covalently via nitrilotriacetic acid (NTA) adaptor, resulting in the display of the drug on the VLNPs (Fig. 1). The pKa of His residue is around 6–6.5 which causes the affinity of His-tag towards NTA-DOX reduces at lower pH due to the protonation of His residues^12–15^. As the pH of intracellular lysosomes and endosomes (pH 5–5.5) is lower than the physiological pH of extracellular fluid (pH 7.4)^8, 9^, this would trigger the release of the attached NTA-DOX from the His-tagged VLNPs.

The His-tagged VLNPs were conjugated with folic acid (FA) using 1-ethyl-3-(3-dimethyl-aminopropyl)-carbodiimide hydrochloride (EDC) and N-hydroxysulfo-succinimide (Sulfo-NHS). These cross-linkers form amide bond between the folate and lysine (Lys) residues at positions 7 and 96 on the HisHBcAg. The FA molecules conjugated to the His-tagged VLNPs would specifically target the particles to cancer cells expressing the folate receptor (FR). FR is highly expressed on the surface of cancer cells (up to 700 times)^16^, including ovary, uterus, kidney, colon, brain and lung cancers as compared with healthy cells^17–19^. In this study, we employed an ovarian carcinoma cell-line, OVCAR-3, expressing high level of FR to demonstrate the feasibility of Add-and-Display of DOX on the His-tagged VLNPs and FR-mediated drug delivery.

Since its first introduction in 1988, the His-tag has been widely used in the production of recombinant proteins to facilitate their detection and purification process^20^. Many viral proteins were also fused with the His-tag and these fusion proteins including the HisHBcAg assembled into VLNPs^10, 21–25^. In the present study, the Add-and-Display approach for displaying a drug non-covalently on VLNPs was established and the drug was released in a controlled manner. This approach would further extend the application of His-tagged molecules in the field of nanomedicine.

## Results

### Synthesis and characterisation of His-tagged VLNPs conjugated with folic acid (FA-HisHBcAg)

**Figure 2:**
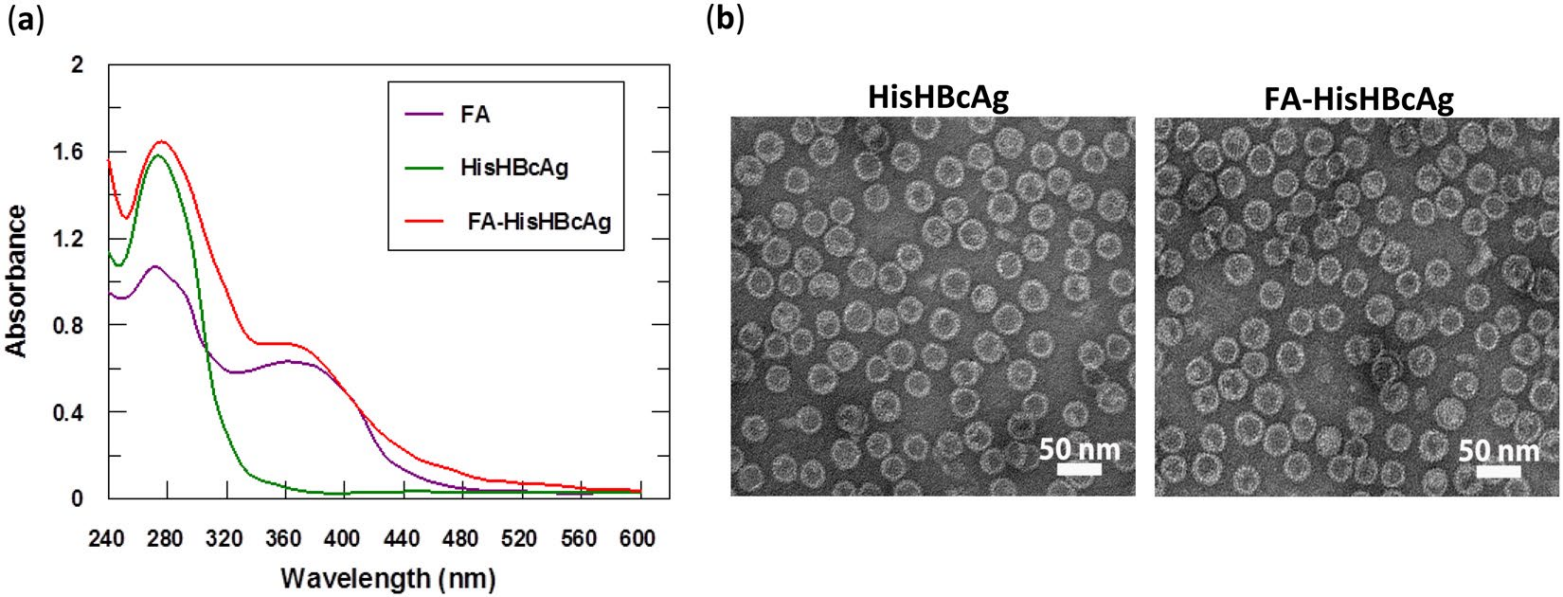
Conjugation of folic acid to His-tagged VLNPs. (**a**) Spectra of folic acid (FA), HisHBcAg nanoparticles (HisHBcAg), and FA-conjugated HisHBcAg nanoparticles (FA-HisHBcAg). (**b**) Electron micrographs of HisHBcAg nanoparticles. The samples (as labelled on top of the micrographs) were stained with uranyl acetate and viewed under a TEM. All the samples assembled into spherical structures. White bars indicate 50 nm.

FA was conjugated to the HisHBcAg nanoparticles by using EDC and Sulfo-NHS. Figure 2a shows the absorbance (A) measurements taken from wavelength 240 to 600 nm. The FA-conjugated HisHBcAg nanoparticles (FA-HisHBcAg) exhibited a significantly higher A_360_ value as compared with the non-conjugated HisHBcAg. About 468 FA molecules were conjugated to each HisHBcAg nanoparticle with a conjugation efficiency (CE_FA_) of 4.1 ± 0.2%. FA:HisHBcAg stoichiometry was determined to be 2:1. Transmission electron microscopy (TEM) revealed that conjugation of FA did not affect the spherical structure of the HisHBcAg nanoparticles (Fig. 2b).

**Figure 3:**
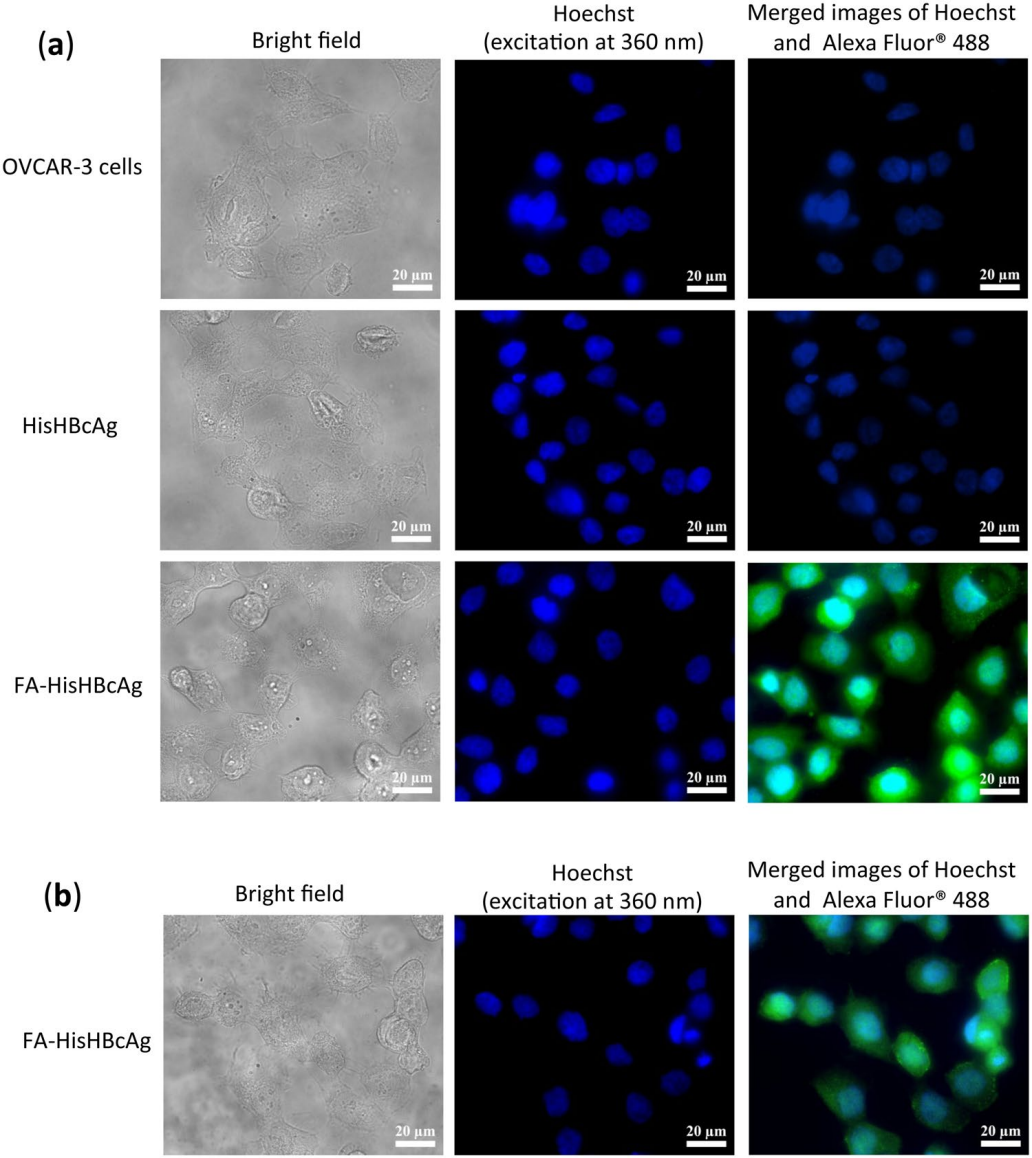
Internalisation of His-tagged VLNPs into OVCAR-3 cells. The HisHBcAg nanoparticles (HisHBcAg; 25 μg) and folic acid (FA)-conjugated HisHBcAg nanoparticles (FA-HisHBcAg; 25 μg) were incubated with OVCAR-3 cells for 16 h at 37 °C. The internalised HisHBcAg nanoparticles were detected by the rabbit anti-HBcAg serum, followed by anti-rabbit antibody conjugated to Alexa Fluor^®^ 488 (**a**), and the mouse anti-His antibody, followed by anti-mouse antibody conjugated to Alexa Fluor^®^ 488 (**b**). Cell nuclei were stained with Hoechst 33342. Non-transfected cancer cells served as a negative control.

The internalisation efficacy of FA-HisHBcAg nanoparticles was studied using OVCAR-3 cells. The HBcAg was detected with the rabbit anti-HBcAg antibody followed by anti-rabbit IgG conjugated to Alexa Fluor^®^ 488 (Fig. 3a), and the His-tag was probed with the anti-His monoclonal antibody followed by anti-mouse IgG conjugated to Alexa Fluor^®^ 488 (Fig. 3b). Immuno-fluorescence microscopy clearly showed that the FA-HisHBcAg nanoparticles translocated into OVCAR-3 cells by emitting bright green fluorescence. The green fluorescence was observed in the cytoplasm of OVCAR-3 cells but it was not detected in the nuclei of the cells labelled with Hoechst (Fig. 3a and b). This demonstrates that internalisation of the FA-HisHBcAg nanoparticles into the OVCAR-3 cells was mediated by FA. The cells incubated with the HisHBcAg nanoparticles which served as a negative control did not emit fluorescence, indicating the HisHBcAg nanoparticles without FA conjugation could not internalise OVCAR-3 cells (Fig. 3a).

### Synthesis and characterisation of His-tagged VLNPs conjugated covalently with FA and non-covalently with NTA-DOX (FA-HisHBcAg-NTA-DOX)

**Figure 4:**
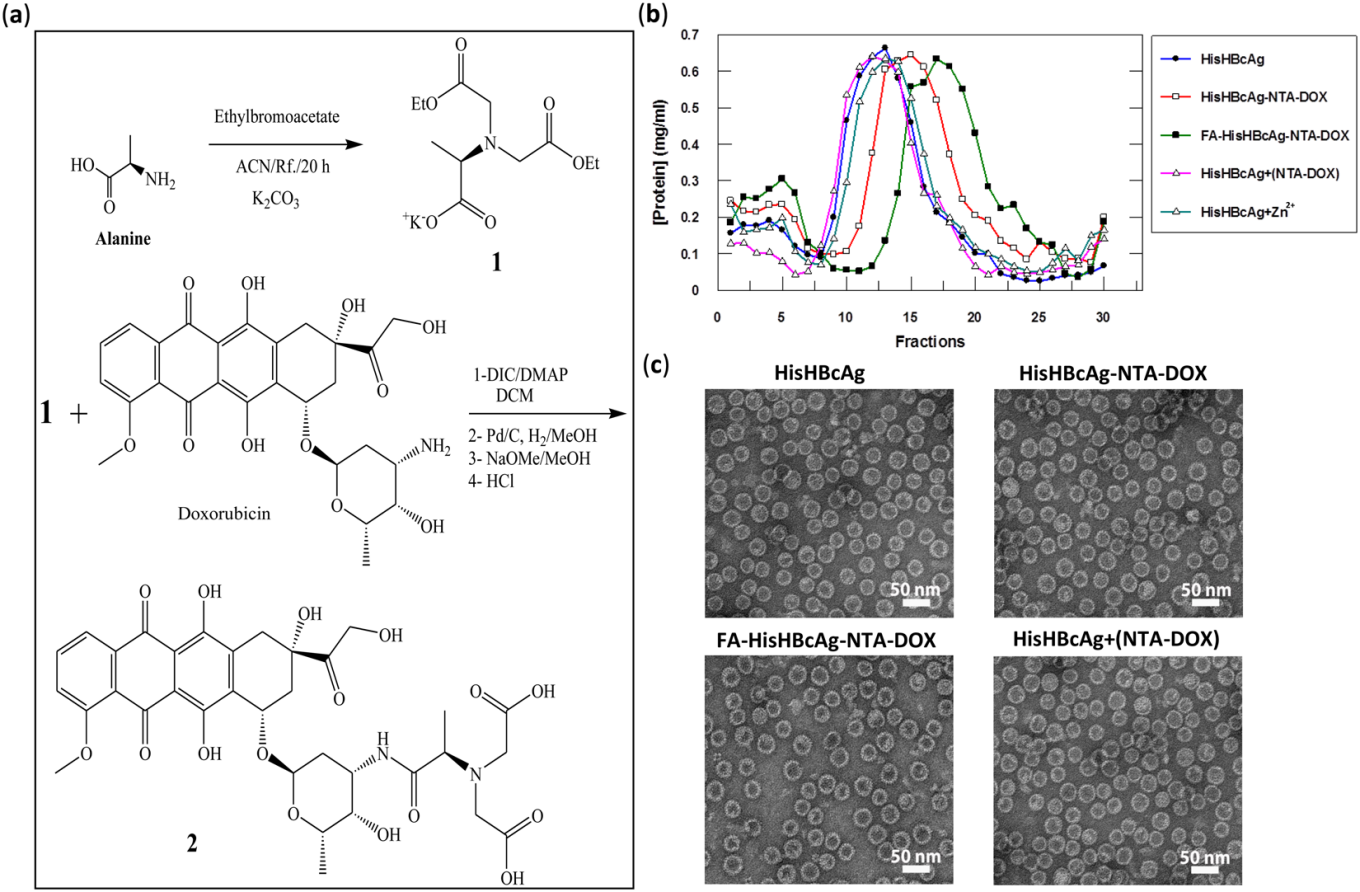
Synthesis and immobilisation of NTA-DOX on His-tagged VLNPs. (**a**) Synthesis of NTA-DOX. The amine group of alanine (Ala) was fully protected with ethylbromoacetate using K_2_CO_3_ under reflux condition to obtain diester-Ala (compound 1). Then, the free carboxylic acid of diester-Ala was activated by N,N′-diisopropylcarbodiimide (DIC) and 4-Dimethylaminopyridine (DMAP). The activated diester-Ala was reacted with doxorubicin (DOX) to produce diester-Ala-DOX, which was then converted to dicarboxylic-Ala-DOX (NTA-DOX; compound 2) by the hydrolysis method using NaOMe in methanol. (**b**) The NTA-DOX was incubated with the HisHBcAg nanoparticles (HisHBcAg) and folic acid (FA)-conjugated HisHBcAg nanoparticles (FA-HisHBcAg) in the presence of Zn^2+^. The nanoparticles conjugated with NTA-DOX were purified by sucrose density gradient ultracentrifugation. The protein amount in each fraction (400 μL) was determined using the Bradford assay. The HisHBcAg nanoparticles (HisHBcAg), HisHBcAg nanoparticles incubated with NTA-DOX in the absence of Zn^2+^ [HisHBcAg + (NTA-DOX)] and HisHBcAg nanoparticles incubated with Zn^2+^ (HisHBcAg + Zn^2+^) served as negative controls. (**c**) Electron micrographs of different HisHBcAg VLNPs formed by HisHBcAg, HisHBcAg-NTA-DOX, FA-HisHBcAg-NTA-DOX, and HisHBcAg + (NTA-DOX). White bars indicate 50 nm.

NTA-DOX containing a His-tag-targeting NTA segment and a DOX segment (Fig. 4a), was synthesised from an NTA-derivative and doxorubicin hydrochloride in a two-step reaction. First, the amine group of alanine (Ala) was fully protected with ethylbromoacetate using K_2_CO_3_ under reflux condition to obtain diester-Ala (compound 1, Supplementary Figure S1). The structure of compound 1 was confirmed using ^1^H and ^13^C nuclear magnetic resonance (NMR) spectroscopy (Supplementary Figure S2). Liquid chromatography-mass spectrometry (LC-MS) analysis of compound 1 revealed a major signal with m/z value of 262.13488 Da (M + H)^+^, which corresponded well with the calculated molecular mass of 261.12 Da (Supplementary Figure S3). Then, compound 1 was mixed with DOX in the presence of 4-Dimethylaminopyridine (DMAP) and N,N’-Diisopropylcarbodiimide (DIC) to synthesise NTA-DOX (Supplementary Figure S4). The structure of NTA-DOX was confirmed using ^1^H and ^13^C NMR (Supplementary Figure S5) and the molecular mass of the NTA-DOX was determined using LC-MS (Supplementary Figure S6). The most abundant ion detected was 731.85509 Da (M + 2H)^+2^, which is in good agreement with the calculated molecular mass of 730.22 Da (Supplementary Figure S6).

The NTA-DOX was incubated with the HisHBcAg and FA-HisHBcAg nanoparticles separately, in the presence of Zn^2+^ and these nanoparticles were analysed with sucrose density gradient ultracentrifugation (Fig. 4b). In the absence of NTA-DOX, the HisHBcAg nanoparticles migrated into the sucrose gradient and accumulated in fractions 9–19. The migration profile is in good agreement with that demonstrated by Yap *et al*.^10^. In the presence of NTA-DOX and Zn^2+^, the HisHBcAg-NTA-DOX and FA-HisHBcAg-NTA-DOX nanoparticles migrated faster in the gradient and accumulated in fractions 11–22 and 13–25, respectively, indicating the presence of denser nanoparticles. Incubation of His-tagged VLNPs with NTA-DOX in the absence of Zn^2+^ [HisHBcAg + (NTA-DOX)] did not show any difference in the migration profile as compared to that of HisHBcAg (Fig. 4b), demonstrating that in the absence of Zn^2+^, NTA-DOX cannot be conjugated to the HisHBcAg nanoparticles. In this experiment, His-tagged VLNP incubated with Zn^2+^ (HisHBcAg + Zn^2+^) was used as a control.

The fractions containing the HisHBcAg-NTA-DOX and FA-HisHBcAg-NTA-DOX nanoparticles were pooled separately, concentrated and viewed under a TEM. Figure 4c shows that the HisHBcAg-NTA-DOX and FA-HisHBcAg-NTA-DOX formed VLNPs morphologically similar with the HisHBcAg nanoparticles. Approximately 622 DOX molecules were conjugated non-covalently to each HisHBcAg nanoparticle with a conjugation efficiency (CE_DOX_) of 7.17 ± 0.40%.

**Figure 5:**
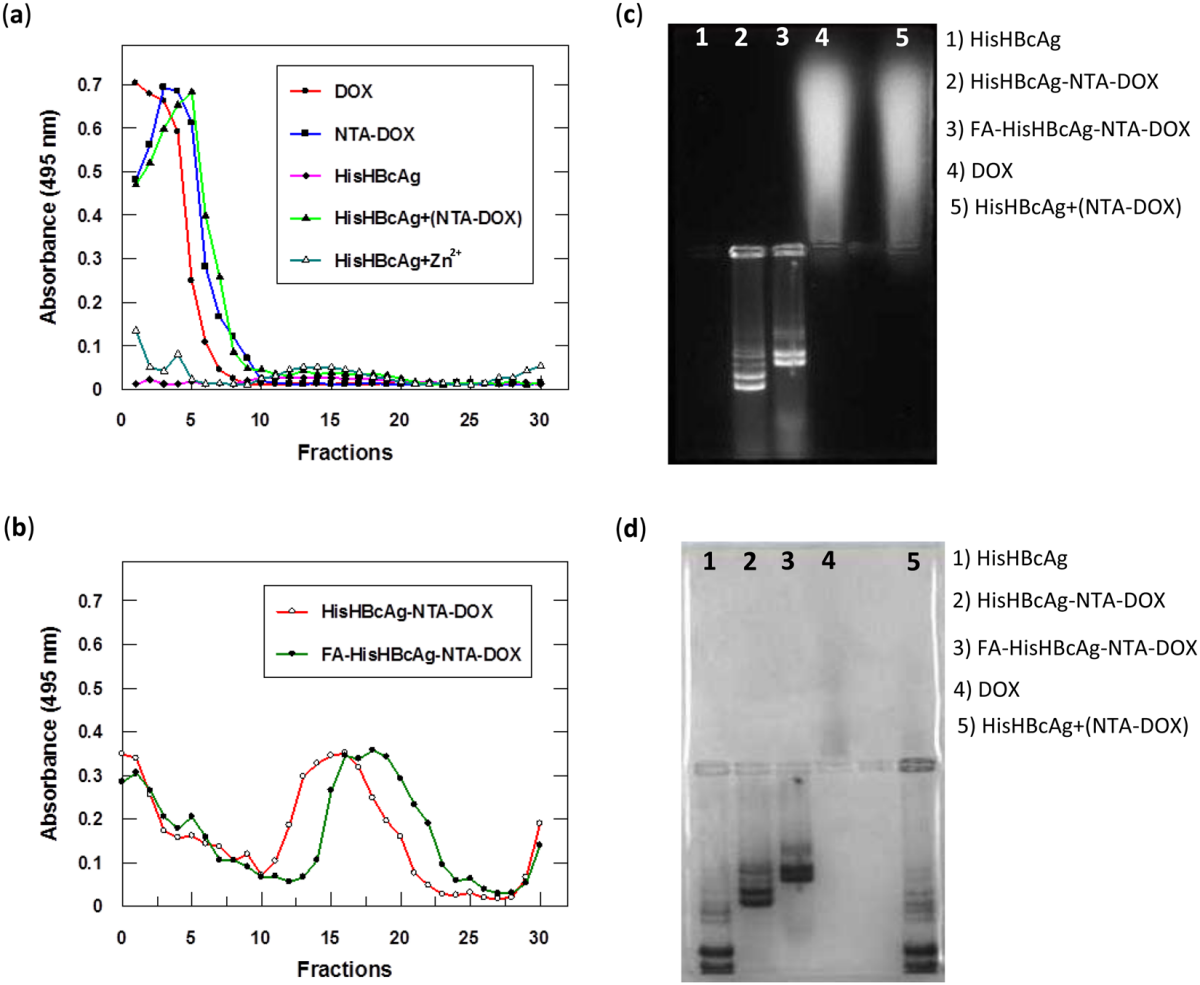
Identification of doxorubicin immobilised on His-tagged VLNPs. (**a** and **b**) Doxorubicin (DOX) was detected by measuring absorbance at 495 nm of the fractions obtained from sucrose gradients. (**a**) DOX, NTA-DOX, HisHBcAg nanoparticles incubated with NTA-DOX in the absence of Zn^2+^ [HisHBcAg + (NTA-DOX)] stayed on top of the sucrose gradients after centrifugation. HisHBcAg nanoparticles (HisHBcAg) were not detected at A_495_. HisHBcAg nanoparticles incubated with Zn^2+^ (HisHBcAg + Zn^2+^) showed a negligible absorbance at 495 nm. (**b**) HisHBcAg nanoparticles conjugated non-covalently with NTA-DOX (HisHBcAg-NTA-DOX) and HisHBcAg nanoparticles conjugated covalently with folic acid (FA) and non-covalently with NTA-DOX (FA-HisHBcAg-NTA-DOX) migrated into the sucrose gradient, and the immobilised DOX was detected with A_495_. (**c** and **d**) Native agarose gel electrophoresis of the HisHBcAg nanoparticles conjugated non-covalently with DOX. The same gel was visualised under (**c**) ultraviolet (UV) illumination, and (**d**) stained with Coomassie Brilliant Blue (CBB).

A_495_ measurement was used to determine the DOX conjugated to the HisHBcAg nanoparticles and their migration profiles in sucrose gradient are shown in Fig. 5a. The result showed that the free DOX, NTA-DOX, and NTA-DOX from HisHBcAg + (NTA-DOX) samples were retained at the top fractions of the gradients (Fig. 5a). The HisHBcAg nanoparticles were not detected at A_495_. The His-tagged VLNP incubated with Zn^2+^ (HisHBcAg + Zn^2+^) showed a negligible absorbance at 495 nm. In contrast, the HisHBcAg-NTA-DOX and FA-HisHBcAg-NTA-DOX were detected in fractions 11–21 and 14–23, respectively (Fig. 5b). The positions of these peaks corresponded well with those of HisHBcAg nanoparticles measured with the Bradford assay (Fig. 4b).

Native agarose gel electrophoresis of intact VLNPs further verified that the particles were successfully conjugated with DOX (Fig. 5c and d). In the electrophoresis, the HisHBcAg nanoparticles migrated towards the anode, while the DOX migrated towards the cathode. The gel visualised under UV light showed that the HisHBcAg-NTA-DOX and FA-HisHBcAg-NTA-DOX nanoparticles migrated to the anode and the protein bands fluoresced, demonstrating that DOX was conjugated to these nanoparticles (Fig. 5c; lanes 2 and 3). The migration of the NTA-DOX incubated with HisHBcAg in the absence of Zn^2+^ [HisHBcAg + (NTA-DOX)] was similar with the free DOX, indicating that NTA-DOX did not bind on the surface of the HisHBcAg nanoparticles in the absence of Zn^2+^. In the gel stained with Coomassie Brilliant Blue (CBB), the HisHBcAg and DOX-conjugated HisHBcAg nanoparticles (HisHBcAg-NTA-DOX) were detected (Fig. 5d). The migration of the non-conjugated HisHBcAg nanoparticles was faster than the conjugated ones (Fig. 5d), demonstrating the conjugation of HisHBcAg nanoparticles with DOX and further with FA significantly reduced the mobility of the VLNPs towards the anode.

### *In vitro* release of doxorubicin from His-tagged VLNPs conjugated covalently with FA and non-covalently with NTA-DOX

**Figure 6:**
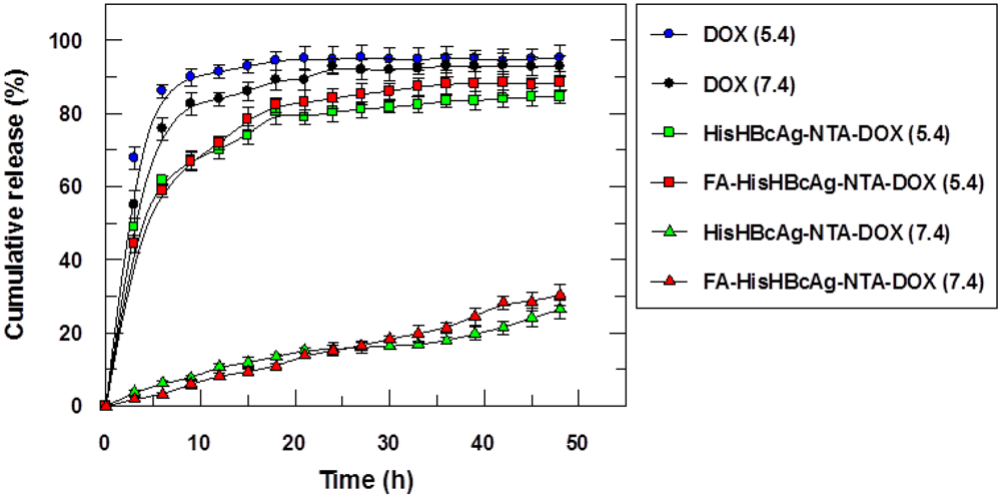
Doxorubicin release profile of the His-tagged VLNPs at different pH. The release profiles of free doxorubicin (DOX), HisHBcAg nanoparticles conjugated non-covalently with NTA-DOX (HisHBcAg-NTA-DOX) and HisHBcAg nanoparticles conjugated covalently with folic acid (FA) and non-covalently with NTA-DOX (FA-HisHBcAg-NTA-DOX) at pH 5.4 and 7.4. More than 80% of the free DOX was released after 5 h at pH 5.4 and pH 7.4, whereas approximately 80% of the conjugated DOX on HisHBcAg nanoparticles was released after 16 h at pH 5.4. Data are expressed as mean ± standard deviation (n = 3).

To study the release behaviour of the HisHBcAg-NTA-DOX and FA-HisHBcAg-NTA-DOX formulations, *in vitro* release experiments were carried out under simulated tumour tissue conditions (pH 5.4, 37 °C) and physiological conditions (pH 7.4, 37 °C). The HisHBcAg-NTA-DOX and FA-HisHBcAg-NTA-DOX formulations exhibited a significant release of DOX at pH 5.4, in which a rapid DOX release appeared instantly upon contacting the release medium, and about 80% of the drug was released at 16 h (Fig. 6). On the other hand, the HisHBcAg-NTA-DOX and FA-HisHBcAg-NTA-DOX formulations exhibited a slow release of DOX at pH 7.4, only about 10% was released at 16 h (Fig. 6). The free DOX diffused rapidly through the dialysis membrane, resulting in 80% cumulative release of DOX after 5 h (Fig. 6). The cumulative release rate of free DOX was not significantly different in both pH solutions.

### Cellular uptake and cytotoxicity of His-tagged VLNPs conjugated covalently with FA and non-covalently with NTA-DOX

**Figure 7:**
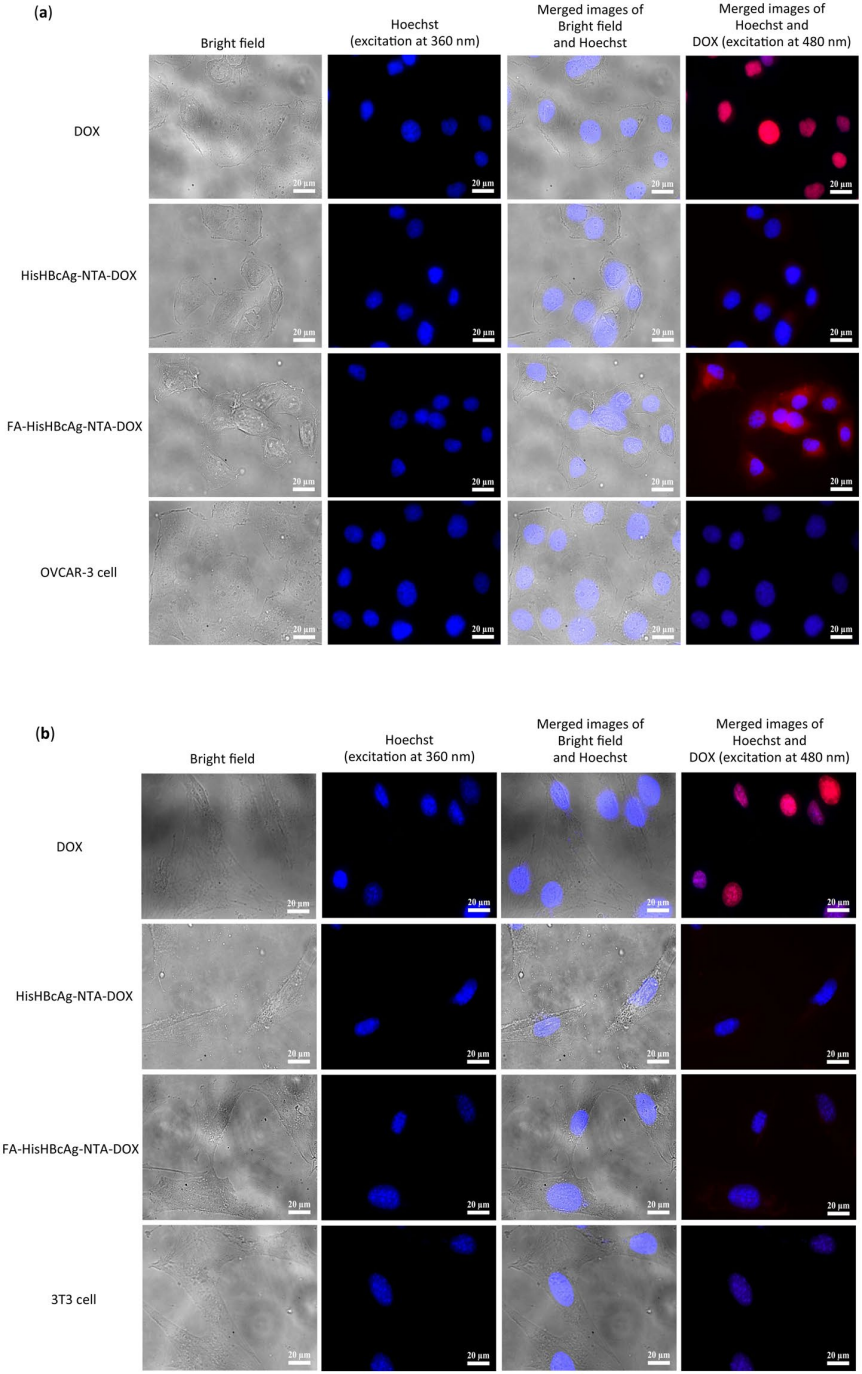
Localisation of His-tagged VLNPs in cancer and normal cells by live cell imaging microscopy. (**a**) Ovarian cancer OVCAR-3 and (**b**) normal 3T3 cells were incubated with free doxorubicin (DOX), HisHBcAg nanoparticles conjugated non-covalently with NTA-DOX (HisHBcAg-NTA-DOX) and HisHBcAg nanoparticles conjugated covalently with folic acid (FA) and non-covalently with NTA-DOX (FA-HisHBcAg-NTA-DOX) at equivalent DOX concentration (5 μg/mL) for 1 h at 37 °C. The untreated cells served as negative controls. Cell nuclei were stained with Hoechst 33342, and DOX was excited at 480 nm and emitted at 535 nm. The samples are labelled on the left. Scale bars indicate 20 μm.

The cellular uptake of free DOX, HisHBcAg-NTA-DOX and FA-HisHBcAg-NTA-DOX nanoparticles in ovarian cancer and normal fibroblast cells was quantified spectrophotometrically and the localisation of these formulations in both cells was studied using live cell imaging microscopy. Quantitative data showed a 3-fold higher uptake of DOX from FA-HisHBcAg-NTA-DOX by OVCAR-3 cells compared with the addition of free DOX (Supplementary Figure S7a). However, the uptake of DOX from HisHBcAg-NTA-DOX and FA-HisHBcAg-NTA-DOX nanoparticles by 3T3 normal cells was lower compared with DOX alone (Supplementary Figure S7b). The cellular uptake of DOX, HisHBcAg-NTA-DOX and FA-HisHBcAg-NTA-DOX nanoparticles was further confirmed by live cell imaging microscopy. OVCAR-3 cells incubated with free DOX exhibited intense red fluorescence and the fluorescence intensity increased significantly when the cells were incubated with the FA-HisHBcAg-NTA-DOX nanoparticles (Fig. 7a). The results of live cell imaging microscopy provide evidence for the FR-mediated uptake of FA-HisHBcAg-NTA-DOX nanoparticles by OVCAR-3 cells. Incubation of 3T3 normal cells with free DOX exhibited a stronger red fluorescent intensity as compared to the same cells incubated with the FA-HisHBcAg-NTA-DOX nanoparticles (Fig. 7b).

**Figure 8:**
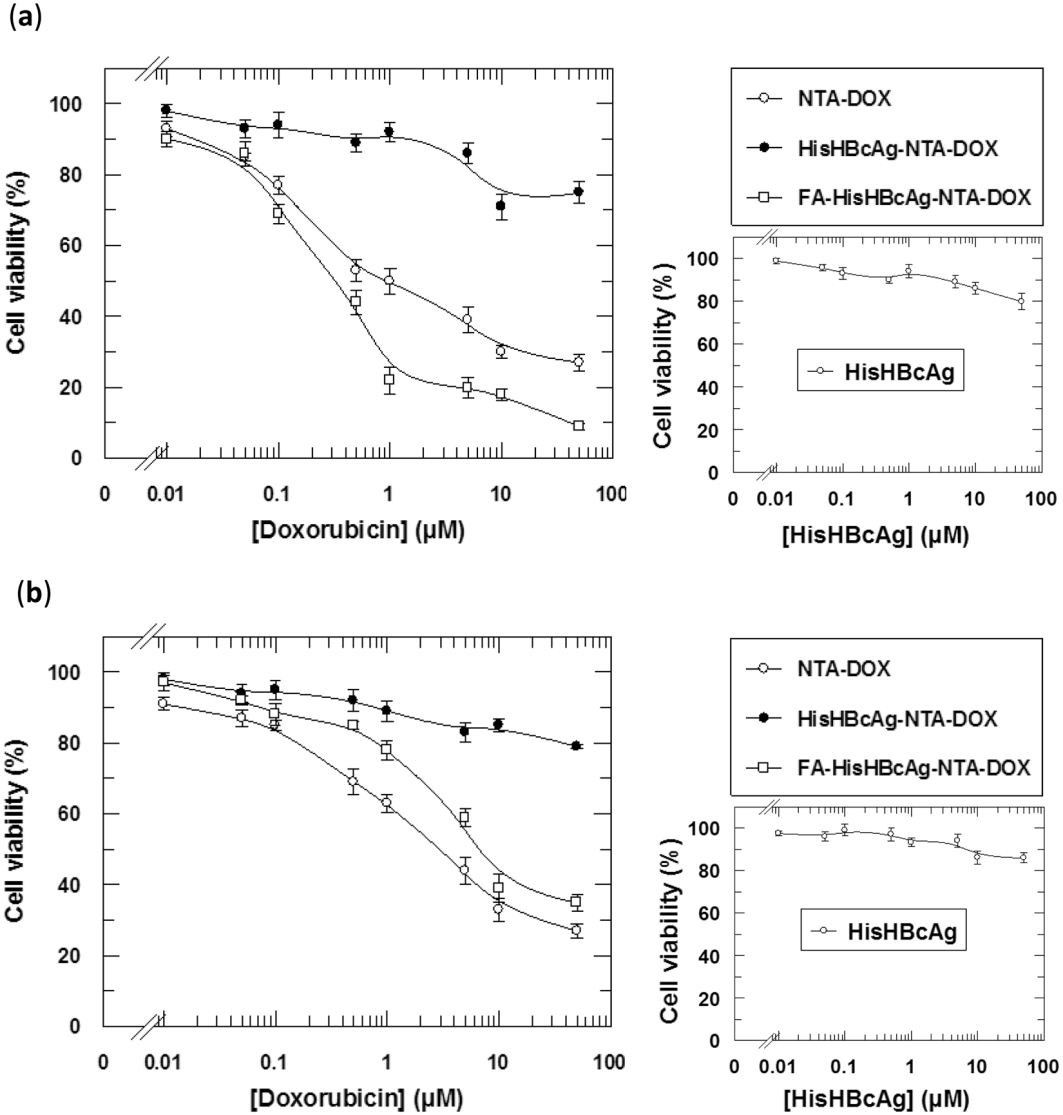
Cytotoxicity analysis of various doxorubicin formulations on ovarian cancer and normal cells. Viability of ovarian cancer OVCAR-3 (**a**), and normal 3T3 (**b**) cells. The HisHBcAg nanoparticles conjugated covalently with folic acid (FA) and non-covalently with NTA-DOX (FA-HisHBcAg-NTA-DOX) were more efficient in inhibiting the growth of OVCAR-3 cells compared to that of other formulations. On the other hand, this formulation (FA-HisHBcAg-NTA-DOX) was less toxic to normal 3T3 cells, resulted in a conferred protection of the normal cells from DOX. The HisHBcAg nanoparticles (HisHBcAg) were not toxic to both normal and cancer cells as shown in the small graphs on the right. Data represent mean ± SD of triplicate determinations.

MTT assay was performed to evaluate the cytotoxicity of various DOX formulations against the OVCAR-3 and 3T3 cells after 3 h of treatment. As compared with free NTA-DOX, the FA-HisHBcAg-NTA-DOX nanoparticles showed a higher cytotoxicity against OVCAR-3 cells (Fig. 8a). The IC_50DOX_ of free NTA-DOX and FA-HisHBcAg-NTA-DOX formulation was 1.00 ± 0.08 μM and 0.31 ± 0.03 μM, respectively (Fig. 8a). The IC_50DOX_ of free NTA-DOX and FA-HisHBcAg-NTA-DOX formulation in 3T3 cells was 2.92 ± 0.28 μM and 6.30 ± 0.30 μM, respectively (Fig. 8b). The HisHBcAg nanoparticles did not show any cytotoxic effect on both the cancer and normal cells.

## Discussion

In conventional drug delivery systems, drugs are either packaged inside or conjugated covalently on the surface of carriers. The packaging and conjugation steps are tedious, laborious and time-consuming. To simplify these steps, a novel Add-and-Display method for immobilisation of cancer drug non-covalently on His-tagged VLNPs was established in this study. The His-tag was employed as a nanojoint to display DOX because many viral proteins including VLNPs produced via recombinant DNA technology are fused with this tag to facilitate protein detection and purification. Most importantly, the His-tag interacts tightly with a transition metal ion and this interaction is reversible by reducing the pH of a surrounding environment. As the contents of intracellular lysosomes and endosomes (pH 5–5.5) are slightly acidic compared to extracellular fluid with a physiological pH 7.4^8, 9, 26^, the protonation of His residues at a lower pH weakens the interaction between the His-tag and metal ions, causing the release of the attached drug, DOX.

DOX was chosen to establish the Add-and-Display method because it is a widely used anti-tumour drug for the treatments of a broad range of human cancers, including breast, ovarian, colon and prostate cancers^15^. However, this drug has several drawbacks such as poor selectivity and high cardiotoxicities^27^. Hence, the major challenge in DOX chemotherapy is to develop a safe and highly effective delivery system, which can deliver the drug into tumour cells with lower systemic toxicity and higher anti-tumour efficiency. In this study NTA-DOX was synthesised from NTA derivative and doxorubicin hydrochloride. The NTA allows the drug to interact with the His-tag via a transition metal, Zn^2+^. By using this approach, approximately 622 DOX molecules were conjugated non-covalently to each HisHBcAg nanoparticle. The HisHBcAg nanoparticles with a triangulation number *T* = 4, is made up of 240 subunits of HBcAg. Cryoelectron microscopy and image reconstruction revealed that the His-tag fused at the N-terminal end of HBcAg monomer formed trimeric spikes and exposed on the surface of the HisHBcAg nanoparticles^11^. Twenty and sixty of these trimeric spikes are located at the icosahedral threefold and fivefold symmetry axes, respectively^11^. Our finding showed that each His-tag associated with 2 to 3 DOX molecules.

The His-tag interacts strongly with Zn^2+^ and Ni^2+^. However, Zn^2+^ was chosen in the present study because several studies conducted in animals and humans demonstrated that zinc could prevent and treat a number of cancers including colorectal, pancreatic, esophageal, head and neck cancers, as well as basal cell carcinoma^28–30^. Zinc is a key constituent or cofactor of over 300 mammalian proteins, which indicates its importance in host defence against the initiation and progression of cancer, therefore it was used in the management and chemoprevention of cancer^28, 29^. Conversely, nickel is hazardous to human body and may cause lung, nasal, sinus, throat and stomach cancers^31^.

pH-responsive nanocarriers have had a significant impact on targeted drug delivery by taking advantage of different pH gradients in extracellular environments and intracellular compartments^6^. Here, we demonstrated that the HisHBcAg nanoparticles conjugated non-covalently with NTA-DOX retained the drug at physiological conditions (pH 7.4, 37 °C), and released the drug in an acidic condition (pH 5.4). The cumulative release of DOX in the tumour tissue conditions was significantly higher (P < 0.01) than that of physiological conditions (pH 7.4, 37 °C). The release of DOX can be explained by protonation of His residues at pH lower than 6.0. The imidazole ring of His has a pKa of ~6^32^, thus at the physiological pH 7.4, the His-tag is mostly deprotonated and uncharged^14^. When the His-HBcAg nanoparticles arrive at acidic environments, such as endosomes and lysosomes, the His residues are protonated, which resulted in the release of DOX.

In order to confer cell-target specificity, FA was conjugated to the Lys residues of HisHBcAg nanoparticles to mediate internalisation of the nanoparticles into tumour cells. Accumulation of DOX in OVCAR-3 cells increased considerably when they were treated with the FA-HisHBcAg-NTA-DOX nanoparticles as compared to free DOX. The result indicated that the FA-HisHBcAg-NTA-DOX nanoparticles bound efficiently to FR, and delivered the drug into FR-positive tumour cells. However, the 3T3 normal cells treated with FA-HisHBcAg-NTA-DOX nanoparticles showed a lower DOX uptake as compared with the free DOX. The higher uptake of FA-HisHBcAg-NTA-DOX by the OVCAR-3 cancer cells compared with the 3T3 normal cells could be due to the fact that the FR is highly expressed in the tumour cells^33, 34^. Furthermore, cancer cells overexpress the α-FR which has a high affinity to the free R-carboxylic acid of the conjugated FA molecules^9, 35, 36^. Whereas, normal cells mostly express the β-FR which has a higher binding affinity for 5-methyltetrahydrofolate, the reduced form of FA^9, 35, 37, 38^. As such, FA conjugated drug-delivery-carrier can internalise the cancer cells at a higher rate compared with normal cells^9^. This is in good accord with our finding in which FA-HisHBcAg-NTA-DOX nanoparticles were taken up at a higher rate by OVCAR-3 cells compared with 3T3 cells.

The cytotoxicity of FA-HisHBcAg-NTA-DOX nanoparticles on OVCAR-3 cells was evaluated by MTT assay. The IC_50DOX_ of FA-HisHBcAg-NTA-DOX nanoparticles (0.31 ± 0.03 μM) was about 3-fold lower compared to that of IC_50DOX_ of free NTA-DOX (1.00 ± 0.08 μM), indicating that the former is more cytotoxic to OVCAR-3 cells compared to the latter. In contrast, the FA-HisHBcAg-NTA-DOX nanoparticles were less toxic to 3T3 cells than free NTA-DOX as the IC_50DOX_ of this formulation (6.30 ± 0.30 μM) increased by approximately 2-fold compared with the IC_50DOX_ of free NTA-DOX (2.92 ± 0.28 μM), demonstrating a conferred protection of the normal cells against the cancer drug.

In summary, a novel Add-and-Display method to conjugate DOX non-covalently on His-tagged VLNPs was established. This method could be used to display other anticancer drugs on His-tagged VLNPs, avoiding laborious and time consuming drug packaging steps. Further, this approach has numerous advantages compared to covalent linkages. It provides facile attachment of drugs on the VLNPs with mild conditions and high coupling efficiencies, without changing the structural conformation of VLNPs.

## Materials and Methods

### Purification of HisHBcAg VLNPs produced in *E. coli*

The HisHBcAg was produced in *E. coli* strain BL21 (DE3) harbouring plasmid pHis-β-L-HBcAg, as described by Yap *et al*.^10^. A fast protein liquid chromatography (FPLC) system (Akta Purifier; GE Healthcare, Sweden) was used to purify the HisHBcAg nanoparticles. The purity of the HisHBcAg was found to be more than 95% pure as analysed with SDS-PAGE. The amount of protein was quantified using the Bradford assay^39^.

### Conjugation of FA to HisHBcAg VLNPs

The carboxylate group of FA was activated by Sulfo-NHS and EDC as described by Biabanikhankahdani *et al*.^9^. The activated carboxyl group was then reacted with the amine group of Lys residues located on the outer surface of HisHBcAg VLNPs, in sodium phosphate buffer (10 mM NaH_2_PO_4_/Na_2_HPO_4_, pH 7.4). The mixture was stirred gently for 8–16 h at 4 °C and separated with sucrose density gradient ultracentrifugation (8–40%) as described by Tan *et al*.^40^. A_360_ of the HisHBcAg nanoparticles (HisHBcAg) and FA-conjugated HisHBcAg nanoparticles (FA-HisHBcAg) was measured using the NanoDrop^TM^ 1000 spectrophotometer (Thermo Scientific, Rockford, IL, USA). The conjugation efficiency of FA (CE_FA_) and the number of FA molecules conjugated to each nanoparticle (N_FA_) were calculated using an extinction coefficient of 5312 mol^−1^cm^−1^, as described by Biabanikhankahdani *et al*.^9^.

### Synthesis of NTA-DOX

Figure 4a summarises the steps used in the synthesis of NTA-DOX. Alanine (Ala; Sigma-Alderich, Louis, MO, USA) was fist reacted with ethylbromoacetate to obtain the diester compound (1). The pH of the reaction mixture was adjusted to 10.5–11.00 using K_2_CO_3_ under reflux condition. The reaction was monitored by thin-layer chromatography (TLC) until the reaction was completed and the amine groups of Ala were fully protected with ethylbromoacetate. Then, the free carboxylic acid of Ala diester was activated by N,N′-diisopropylcarbodiimide (DIC) and 4-Dimethylaminopyridine (DMAP). The activated diester-Ala was reacted with DOX (Merck Millipore, Billerica, MA, USA) under controlled pH. The pH of reaction must be in the range of 8–8.5. Diester-Ala-DOX was converted to the dicarboxylic-Ala-DOX compound by hydrolysis method using NaOMe in methanol. The obtained product was purified with silica gel chromatography using MeOH:CHCl_3_ (2:8) as the mobile phase (supplementary information).

### Immobilisation of NTA-DOX on HisHBcAg VLNPs

The HisHBcAg VLNPs (50 nM) were mixed with NTA-DOX (15 μM) and ZnCl_2_ (20 μM). The mixture was incubated at room temperature for 4 h, and at 4 °C overnight. The mixture was then dialysed against dialysis buffer (50 mM Tris-HCl, 100 mM NaCl, pH 8.0; 1 L, two times) at 4 °C. The HisHBcAg and FA-HisHBcAg nanoparticles conjugated with NTA-DOX were purified as described by Tan *et al*.^40^. The samples (500 μL) were applied on sucrose density gradient (8–40%, w/v), centrifuged (210,000 × g, for 5 h at 4 °C), and fractionated (400 µL per fraction). The protein concentration in each fraction was analysed with the Bradford assay^39^ while the amount of DOX was measured at A_495_. Sucrose fractions containing the HisHBcAg-NTA-DOX and FA-HisHBcAg-NTA-DOX nanoparticles were then pooled, dialysed in dialysis buffer and the morphology of the nanoparticles was observed under a TEM. In this experiment, the HisHBcAg nanoparticles (HisHBcAg) and the HisHBcAg nanoparticles added with NTA-DOX in the absence of Zn^2+^ [HisHBcAg + (NTA-DOX)] were also purified on sucrose density gradient and used as controls. The conjugated DOX was quantified at A_495_ using an extinction coefficient of 8030 cm^−1^M^−1^. The conjugation efficiency of DOX (CE_DOX_) and the number of DOX conjugated to each nanoparticle (N_DOX_) were calculated using equations 1 and 2, respectively.

1 $${{\rm{CE}}}_{{\rm{DOX}}} \% ={{\rm{weight}}}_{{\rm{DOX}}}/{{\rm{weight}}}_{{\rm{HisHBcAg}}{\rm{particle}}}\times 100 \% $$

2 $${{\rm{N}}}_{{\rm{D}}{\rm{O}}{\rm{X}}}={{\rm{C}}{\rm{E}}}_{{\rm{D}}{\rm{O}}{\rm{X}}}\times ({{\rm{M}}{\rm{w}}}_{{\rm{H}}{\rm{i}}{\rm{s}}{\rm{H}}{\rm{B}}{\rm{c}}{\rm{A}}{\rm{g}}{\rm{p}}{\rm{a}}{\rm{r}}{\rm{t}}{\rm{i}}{\rm{c}}{\rm{l}}{\rm{e}}}/{{\rm{M}}{\rm{w}}}_{{\rm{D}}{\rm{O}}{\rm{X}}})$$

Mw represents the molecular weight.

### Cell culture

The human ovarian epithelial adenocarcinoma (OVCAR-3) cells were obtained from the American Type Culture Collection (ATCC) and the normal fibroblast cells (3T3) were provided by the Laboratory of Molecular Biomedicine, Universiti Putra Malaysia. The cell lines were cultured in FA-deficient RPMI1640 medium (Gibco Life Technology, Grand Island, NY, USA) continuously as a monolayer supplemented with heat-inactivated fetal bovine serum (FBS, 10%), at 37 °C in a humidified atmosphere of 5% CO_2_. The cells were passaged twice weekly.

### Transmission electron microscopy (TEM)

The HisHBcAg nanoparticles and derivatives (~0.25 mg/mL; 15 μL) were coated onto carbon coated grids (200 mesh). The particles were stained negatively with freshly prepared uranyl acetate [2% (w/v)] for 5 min. The grids were observed under a TEM (100 kV; Hitachi H7700, Japan).

### Internalisation of HisHBcAg VLNPs into OVCAR-3 cells

The internalisation of FA-conjugated HisHBcAg nanoparticles into OVCAR-3 cells was detected using the anti-His monoclonal antibody and the anti-HBcAg serum. Different samples of HisHBcAg nanoparticle (25 μg) were added to the cells seeded in a six-well plate (200,000 cells/well), and incubated for 16 h at 37 °C. The cells were washed and fixed with paraformaldehyde (3.7%) in phosphate buffered saline [PBS; 137 mM NaCl, 2.7 mM KCl, 1.47 mM KH_2_PO_4_, 8.1 mM Na_2_HPO_4_; pH 7.4] for 10 min. After permeabilising the cells with ice-cold methanol at −20 °C for 6 min, the cells were incubated in blocking buffer (0.2 mg/mL BSA in PBS) for 1 h to block nonspecific binding. The cells were incubated with the anti-His monoclonal antibody (1:3000 dilutions) or the rabbit anti-HBcAg serum (1:200 dilutions) for 1 h at room temperature. Then, the goat anti-mouse IgG conjugated to Alexa Fluor® 488 (1:1000 dilutions in blocking buffer; Abcam, Cambridge, UK) or goat anti-rabbit IgG conjugated to Alexa Fluor^®^ 488 (1:1000 dilutions in blocking buffer; Life Technologies, Carlsbad, CA, USA) was added to the cells and incubated for 1 h in dark. The cell nuclei were stained with Hoechst 33342 (Ex_360 nm_ and Em_460 nm_; Life Technologies, Eugene, OR, USA) for 15 min, and observed under the Olympus Live Cell Imaging (Center Valley, PA, USA). In this experiment, the untreated cells and the cells added with the HisHBcAg nanoparticles were used as negative controls.

### Native agarose gel electrophoresis (NAGE)

The migration profiles of the HisHBcAg nanoparticles conjugated with NTA-DOX and FA were examined with NAGE. The samples were electrophoresed on a native agarose gel as described by Biabanikhankahdani *et al*.^9^ and Yoon *et al*.^41^. DOX bands were visualised by ultraviolet illumination (UV) using the GelDoc 2000 Imaging System (Bio-Rad, Philadelphia, PA, USA), while proteins were stained with CBB.

### *In vitro* release of DOX from HisHBcAg VLNPs

Drug release experiment was performed by the dialysis method^9, 33^ with some modifications. DOX, HisHBcAg-NTA-DOX and FA-HisHBcAg-NTA-DOX samples (1 mL; equivalent to 250 μg/mL DOX) were placed in individual dialysis tubes (MWCO 12 KDa; Sigma-Aldrich, Louis, MO, USA). The samples were dialysed against 40 mL of PBS (pH 7.4 and pH 5.4, separately) with gentle and constant stirring at 37 °C. To quantify the released DOX, 1 mL of the release buffer was collected at specific time point and A_495_ was measured. For each collection, the collected buffer was replaced with the same volume of fresh medium.

### Localisation of HisHBcAg VLNPs in normal and cancer cells

OVCAR-3 and 3T3 cells were subcultured in six well plates at 2.0 × 10^5^ and 1.0 × 10^5^ cell/mL, respectively, and incubated for 24 h. After that, each medium was replaced with fresh medium containing free DOX, HisHBcAg-NTA-DOX or FA-HisHBcAg-NTA-DOX (1 mL; with a constant concentration of 5 μg/mL DOX). The cells were incubated for 1 h, washed and fixed with 3.7% (w/v) paraformaldehyde in PBS (pH 7.4) for 10 min at 25 °C. Cell nuclei were stained with Hoechst 33342. The stained substrates were imaged by using the Olympus Live Cell Imaging (EX_480 nm_ and Em_535 nm_; Center Valley, PA, USA). In this experiment, the HisHBcAg nanoparticles and untreated cells served as negative controls.

### Cytotoxicity of HisHBcAg VLNPs conjugated with FA and NTA-DOX

MTT assay was used to examine the cytotoxicity of NTA-DOX, HisHBcAg-NTA-DOX and FA-HisHBcAg-NTA-DOX nanoparticles on ovarian cancer OVCAR-3 cells and normal fibroblast 3T3 cells. OVCAR-3 and 3T3 cells (1 × 10^4^ and 7 × 10^3^ cells/well, respectively) were seeded in 96-well plates and incubated for 24 h. After aspirating the media, different concentrations of free NTA-DOX or DOX formulations in media (0.01–50 μM) were added to each well for 3 h. The old media containing NTA-DOX or different formulations were exchanged with fresh media. The cells were incubated for 72 h and then the MTT solution was added. After 4 h of incubation, A_570_ was measured using the Uquant ELISA plate reader (BioTeck Instruments, Winooski, VT, USA). The cytotoxicity of HisHBcAg nanoparticles was studied as a negative control.

### Statistical analysis

Statistical analysis was performed using the SPSS programme. Values of p < 0.01 are considered to be significant.

## Electronic supplementary material

Supplementary Information
