# Supplementary material for: A Simple Add-and-Display Method for Immobilisation of Cancer Drug on His-tagged Virus-like Nanoparticles for Controlled Drug Delivery
Source: Sci Rep. 2017 Jul 13;7:5303. doi: 10.1038/s41598-017-05525-4 (PMC5509718; doi:10.1038/s41598-017-05525-4)
Supplement: Supplementary file 1 — Supplementary Information [file 41598_2017_5525_MOESM1_ESM.pdf]

Supplementary Information

A Simple Add-and-Display Method for Immobilisation of Cancer Drug on His-tagged Virus-like Nanoparticles for Controlled Drug Delivery

Roya Biabanikhankahdani[1], Saadi Bayat[2], Kok Lian Ho[3], Noorjahan Banu Mohamed Alitheen[4,5], Wen Siang Tan[*1,5]

[1]Department of Microbiology, Faculty of Biotechnology and Biomolecular Sciences, Universiti Putra Malaysia, 43400 UPM Serdang, Selangor, Malaysia.

[2]Department of Chemistry, Faculty of Science, Universiti Putra Malaysia, 43400 UPM Serdang, Selangor, Malaysia.

[3]Department of Pathology, Faculty of Medicine and Health Sciences, Universiti Putra Malaysia, 43400 UPM Serdang, Selangor, Malaysia.

[4]Department of Cell and Molecular Biology, Faculty of Biotechnology and Biomolecular Sciences, Universiti Putra Malaysia, 43400 UPM Serdang, Selangor, Malaysia.

[5]Institute of Bioscience, Universiti Putra Malaysia, 43400 UPM Serdang, Selangor, Malaysia

Synthesis and Analysis of NTA-DOX

1. Synthesis of compound 1

Alanine (0.025 mmol; Sigma-Aldrich) in acetonitrile (ACN, 30 mL) was added with K_2CO_3 (2 g) and ethylbromoacetate (0.03 mmol). The mixture was refluxed for 20 h and the reaction is shown in Supplementary Figure S1. The reaction progress was monitored using thin-layer chromatography (TLC; Et:n-Hex, 1:1) on silica gel plates (60F-254) and visualized using $KMnO_4$. The reaction was completed after 20 h. Then, the solvent was evaporated under vacuum using a rotary evaporator. The product was extracted and purified using EtOAc (2×30 mL) and water (30 mL). The organic layer was then washed with brine and water. The organic layer was

dried with Na_2SO_4 and evaporated to obtain precipitates of compound **1**. The yield of the product was 95%.

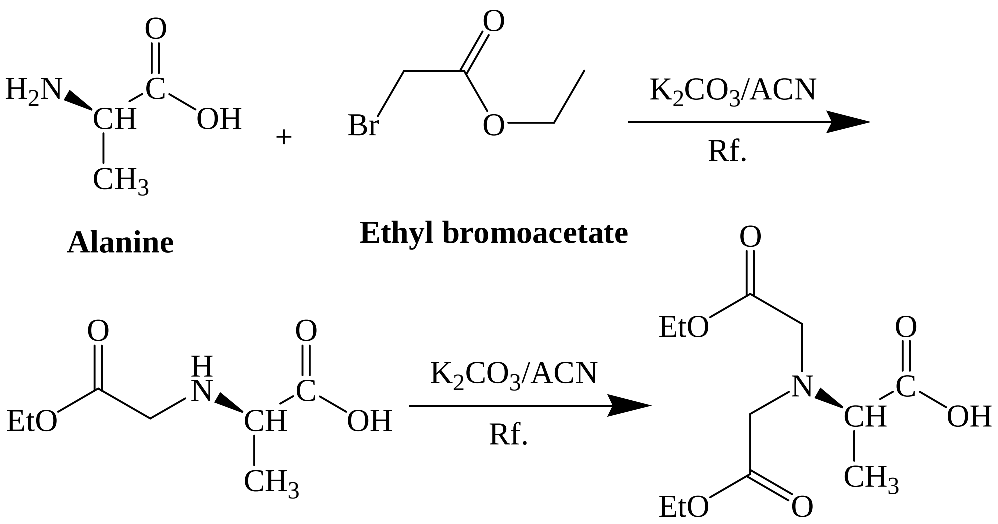

Mono Protected

(1)
Fully Protected Amine Group

Supplementary Figure S1. Synthesis of fully protected amine group of alanine. ACN, acetonitrile; K_2CO_3, potassium carbonate; Rf., reflux.

Compound 1 was analysed using a nuclear magnetic resonance (NMR) spectrophotometer (Varian, 500 MHz). All ^{1}H and ^{13}C NMR spectra (Supplementary Figure S2) were recorded at 25°C in methanol-d4. Chemical shifts (δ) were reported in parts per million (ppm). Multiplicities are abbreviated as follows: s = singlet, d = doublet, t = triplet, q = quartet, m = multiplet.

(a)

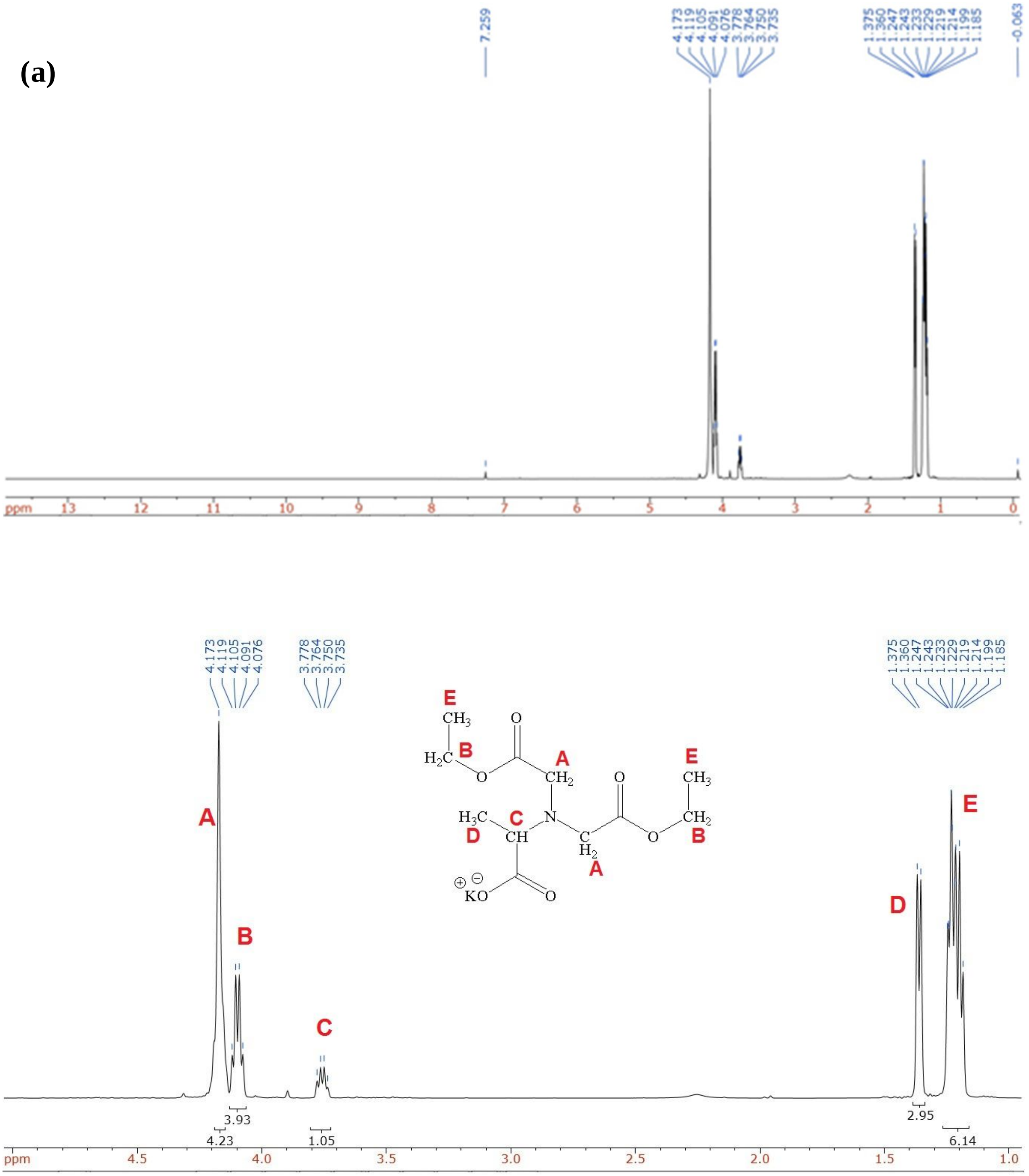

7.259
4.173
4.119
4.105
4.091
4.076
3.778
3.764
3.750
3.735
1.375
1.360
1.247
1.243
1.233
1.229
1.219
1.214
1.199
1.185
-0.063
ppm 13 12 11 10 9 8 7 6 5 4 3 2 1 0
A
B
C
D
E
3.93
4.23
1.05
2.95
6.14
ppm 4.5 4.0 3.5 3.0 2.5 2.0 1.5 1.0

(b)

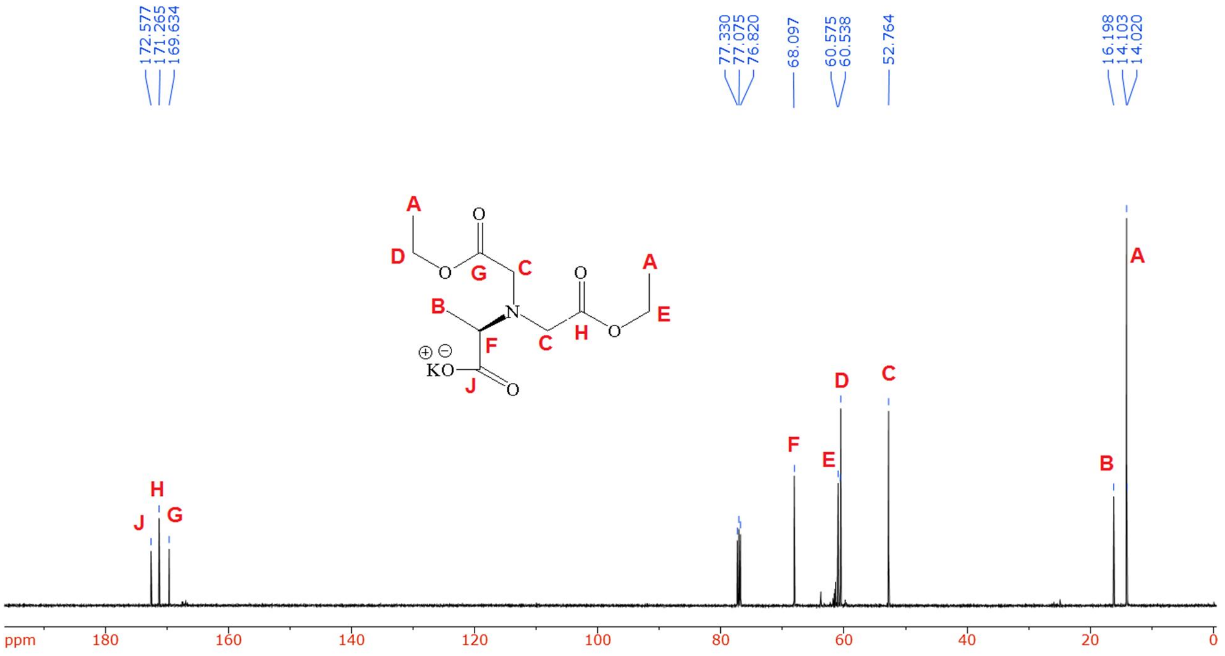

Supplementary Figure S2. NMR spectroscopy of compound 1. (**a**) ^{1}HNMR, (**b**)^{13}CNMR spectra of compound 1.

^{1}HNMR (500 MHz, $CDCl_3$), δ (ppm) = 4.17(s, 4H, A), 4.09(q, 4H, J = 7.14 Hz, B), 3.75(q, 1H, J = 7.0 Hz, C), 1.36(d, 3H, J = 7.5 Hz, D), 1.20 – 1.22(m, 6H, E).

^{13}CNMR (100 MHz, $CDCl_3$), δ (ppm) = 14.02, 14.10, 16.19, 52.79, 60.53, 60.57, 68.09, 169.63, 171.26, 172.57.

Compound 1 was analysed using a LC-MS (Agilent 1290 Infinity LC system coupled to Agilent 6520 Accurate-Mass Q-TOF mass spectrometer with dual ESI source) and the data are shown in Supplementary Figure S3.

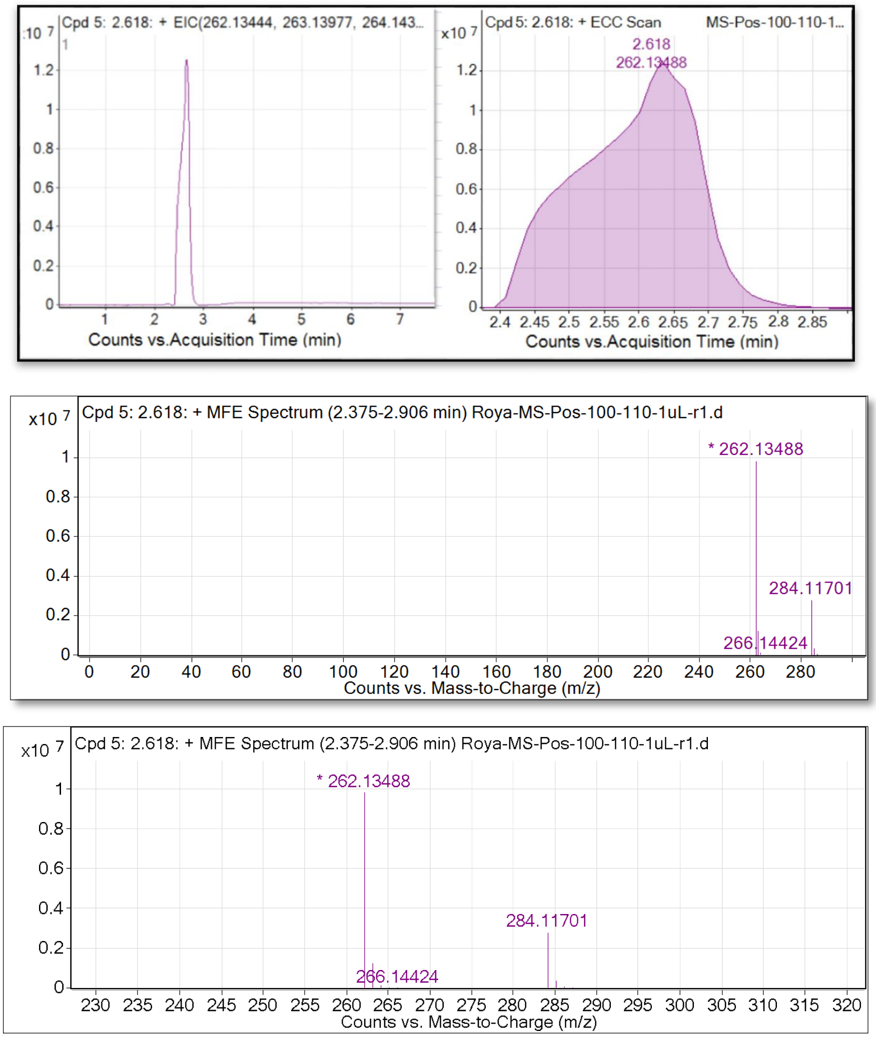

MS Spectrum Peak List

m/z	z	Abundance	Ion
262.13488	1	9841889.00	$(M+H)^+$
263.13813	1	1222952.75	$(M+H)^+$
264.13983	1	111440.54	$(M+H)^+$
265.14235	1	9659.17	$(M+H)^+$
266.14424	1	0	$(M+H)^+$
284.11701	1	2789810.25	$(M+Na)^+$
285.12010	1	364496.28	$(M+Na)^+$
286.12157	1	55474.67	$(M+Na)^+$
287.12385	1	4384.43	$(M+Na)^+$

Supplementary Figure S3. Mass spectroscopy of compound 1. Molecular mass was reported as $(M+H)^+$ and $(M+Na)^+$. The most abundant ions detected were 262.13488 Da $(M+H)^+$ and 284.11701 Da $(M+Na)^+$. The calculated molecular mass by the Chemdraw software was 261.12 Da.

2. Synthesis of compound 2

Compound 1 (4.5 mg) in dichloromethane (DCM, 10 mL) was added with doxorubicin (DOX, 5 mg) and 4-Dimethylaminopyridine (DMAP, 5 mg). The mixture was kept in ice-bath for 30 minutes. Then, a solution of N,N′-Diisopropylcarbodiimide (DIC, 5 mg) in DCM (3 mL) was added to the above mixture and stirred for 3 h at room temperature (RT). Completion of the reaction was checked by TLC ($CHCl_3$:MeOH, 9:1) and visualized under UV light. The reaction completed after an overnight incubation. The mixture was filtered and the filtrate was evaporated under vacuum using a rotary evaporator. The product was extracted and purified using EtOAc (2×30 mL) and water (30 mL). The organic layer was then washed with brine and water. The organic layer was dried with Na_2SO_4 and evaporated. The crude compound was purified using a flash chromatography ($CHCl_3$:MeOH, 8:2) and the yield of the product was 95%.

A solution of NaOMe (28%, 20 µL) was added to a solution of above deprotected compound (3 mg) in 60% aqueous CH_3OH (5 mL) and stirred for 1 h at RT. The reaction is depicted in Supplementary Figure S4. When the reaction was completed, the pH of the mixture was adjusted to 6-7 by adding 0.1 M HCl. Then the solvent was desalted using an ion exchange resin (Dowex 50WX8-200). The solvent was evaporated using a rotary evaporator under reduced pressure. The crude product was purified using flash column chromatography on silica gel (CH_3Cl:CH_3OH 8:2) to obtain compound **2** (NTA-DOX) with a yield of 85%.

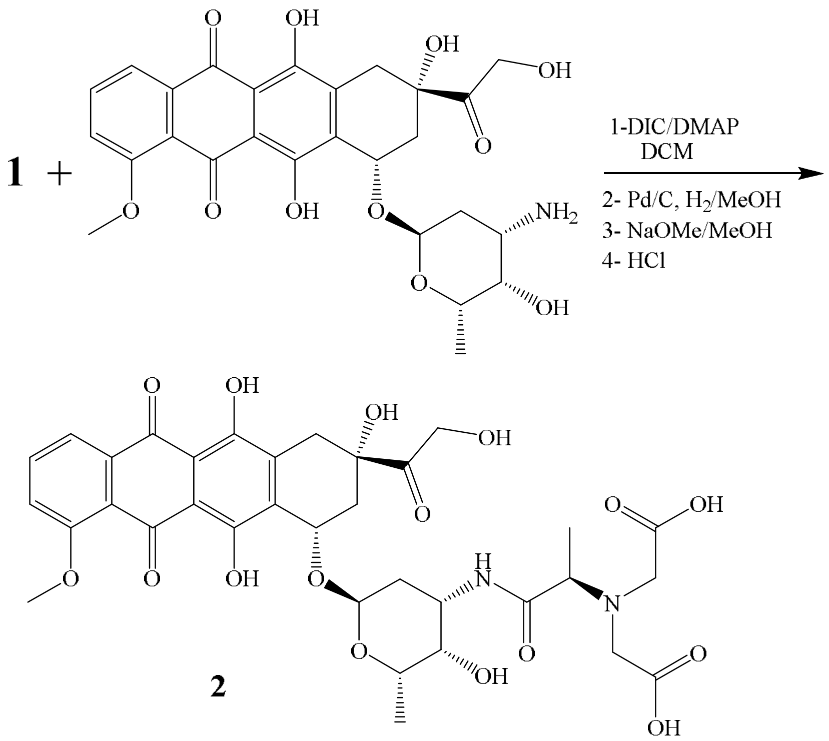

Supplementary Figure S4. Synthesis of NTA-DOX. Compound 1 in dichloromethane (DCM) was mixed with doxorubicin (DOX) in the presence of 4-Dimethylaminopyridine (DMAP) and *N,N'*-Diisopropylcarbodiimide (DIC) at RT for 3 h. Then the product was converted to the dicarboxylic-Ala-DOX (NTA-DOX) compound by the hydrolysis method using NaOMe in methanol.

The synthesised NTA-DOX was analysed using a nuclear magnetic resonance (NMR) spectrophotometer (Varian, 500 MHz). All ^{1}H and ^{13}C NMR spectra (Supplementary Figure S5) were recorded at 25°C in methanol-d4. Chemical shifts (δ) were reported in parts per million (ppm). Multiplicities are abbreviated as follows: s = singlet, d = doublet, t = triplet, q = quartet, m = multiplet.

(a)

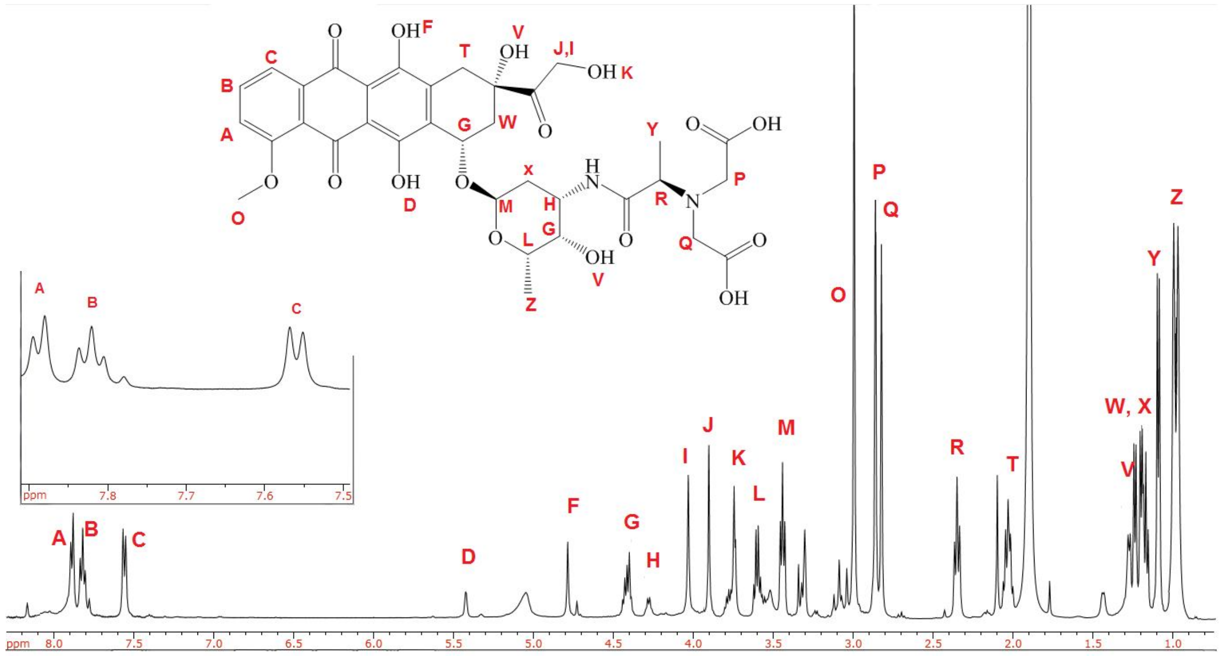

A
B
C
D
F
G
H
I
J
K
L
M
O
P
Q
R
T
V
W, X
Y
Z
OH
O
H
N
OH
ppm
8.0
7.5
7.0
6.5
6.0
5.5
5.0
4.5
4.0
3.5
3.0
2.5
2.0
1.5
1.0
7.8
7.7
7.6
7.5

(b)

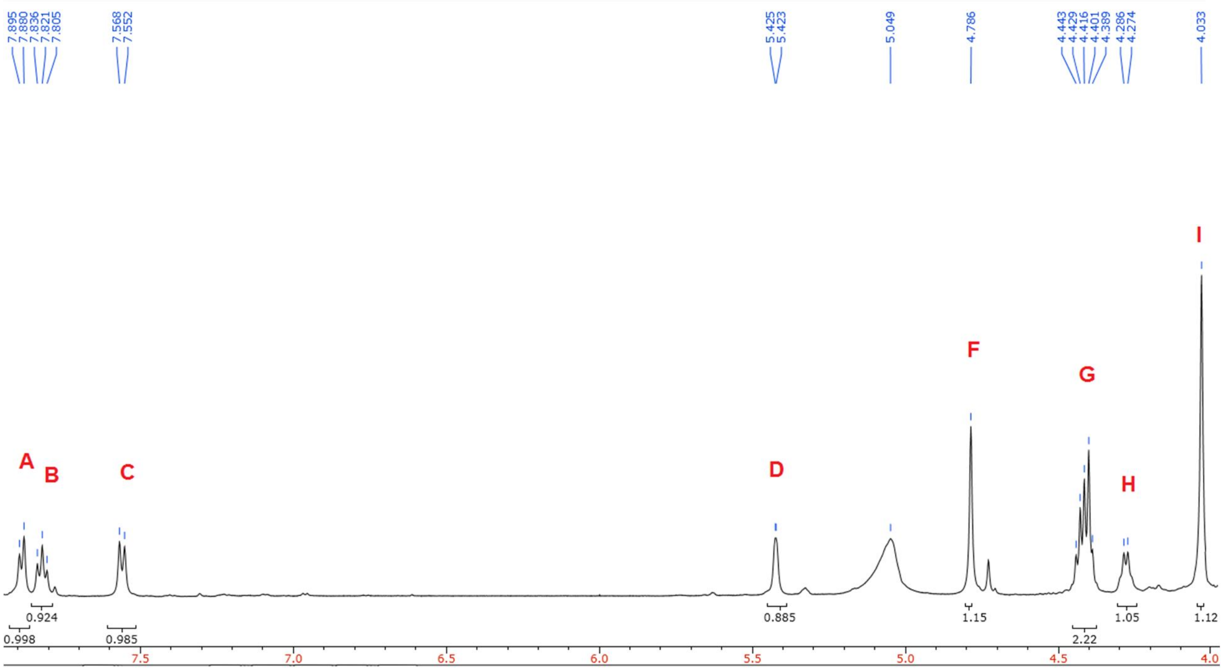

7.895
7.880
7.836
7.821
7.805
7.568
7.552
5.425
5.423
5.049
4.786
4.443
4.429
4.416
4.401
4.389
4.286
4.274
4.033
A
B
C
D
F
G
H
I
0.998
0.924
0.985
0.885
1.15
2.22
1.05
1.12
7.5
7.0
6.5
6.0
5.5
5.0
4.5
4.0

(c)

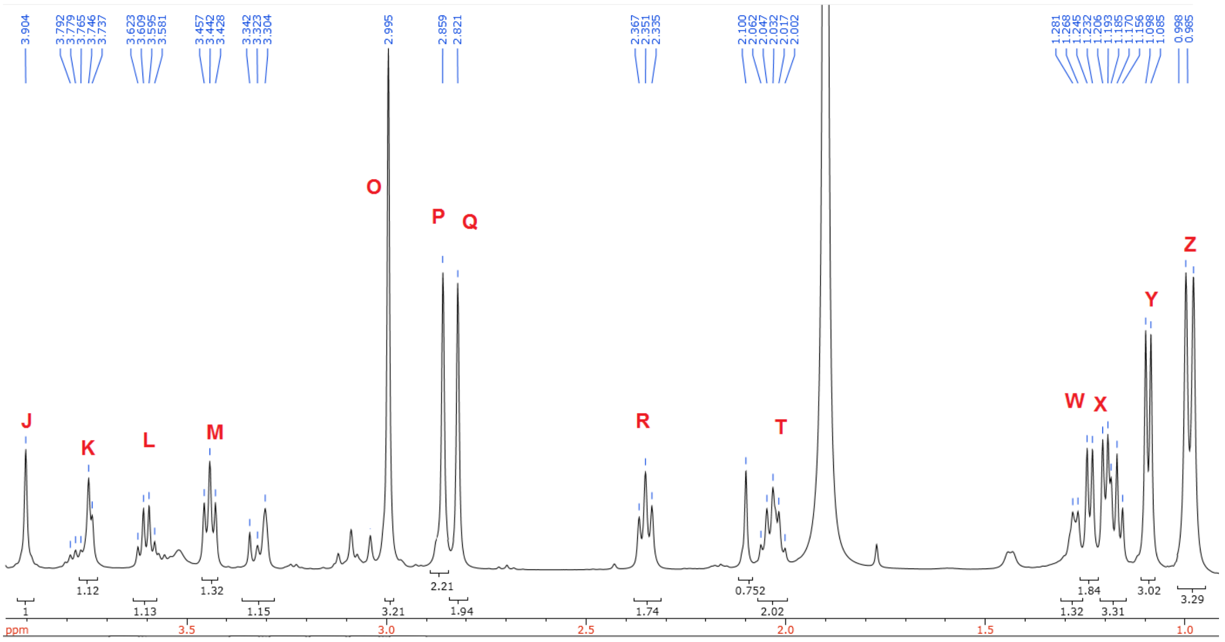

(d)

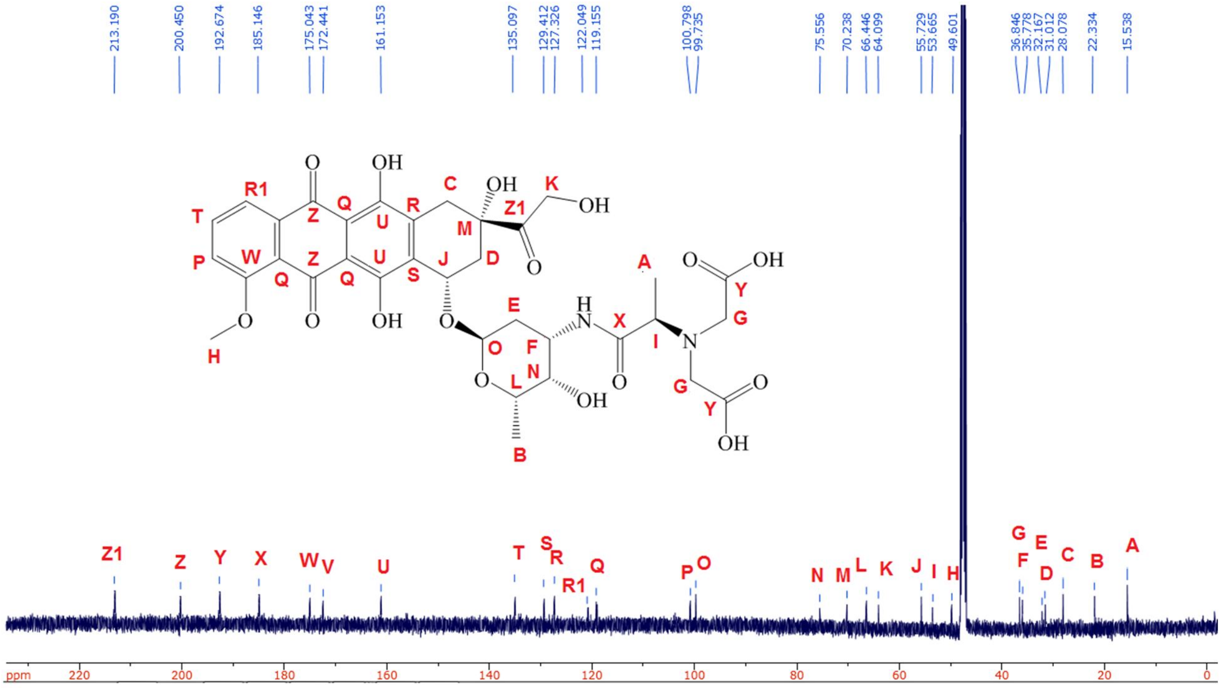

Supplementary Figure S5. ^{1}HNMR and ^{13}CNMR spectra of NTA-DOX. (**a**, **b**, and **c**) ^{1}HNMR spectra of NTA-DOX, (**d**) ^{13}CNMR of NTA-DOX.

^{1}HNMR (500 MHz, CD_3OD), δ 7. 8(d, 1H, J = 7.38 Hz, A), 7.82(t, 1H, J = 7.87 Hz, B), 7.56(d, 1H, J = 8.25 Hz, C), 5.42(d, 1H, J = 1.1 Hz, D), 4.78(s, 1H, F), 4.41(dt, 2H, J = 13.67, 6.67 Hz, G), 4.27(d, 1H, J =6.43 Hz, H), 4.03(s, 3H, I) 3.90(s, 1H, J), 3.76(s, 1H, K), 3.60(q, 1H, J = 7.00 Hz, L), 3.44(t, 1H, J = 7.06 Hz, M), 3.06(s, 3H, O), 2.99(s, 2H, P), 2.82(s, 2H, Q), 2.35(t, 2H, J = 8.04 Hz, R), 2.03(dt, 2H, J = 15.04, 7.54 Hz, T), 1.43(d, J = 5.59 Hz, U), 1.27(d, 1H, J = 6.59Hz, V), 1.23-1.18(m, 3H, W), 1.09(d, 3H, J = 6.47Hz, X), 0.99(d, 3H, J = 8.56Hz, Y)

^{13}CNMR (100 MHz, CD_3OD), 15.53, 22.33, 28.07, 31.01, 32.16, 35.77, 36.84, 49.60, 53.66, 55.72, 64.09, 66.44, 70.23, 75.55, 99.73, 100.79, 119.15, 122.04, 127.32, 129.41, 135.09, 161.15, 172.44, 175.04, 185.14, 192.67, 200.45, 213.19.

NTA-DOX was analysed using a LC-MS (Agilent 1290 Infinity LC system coupled to Agilent 6520 Accurate-Mass Q-TOF mass spectrometer with dual ESI source) and the data are shown in Supplementary Figure S6.

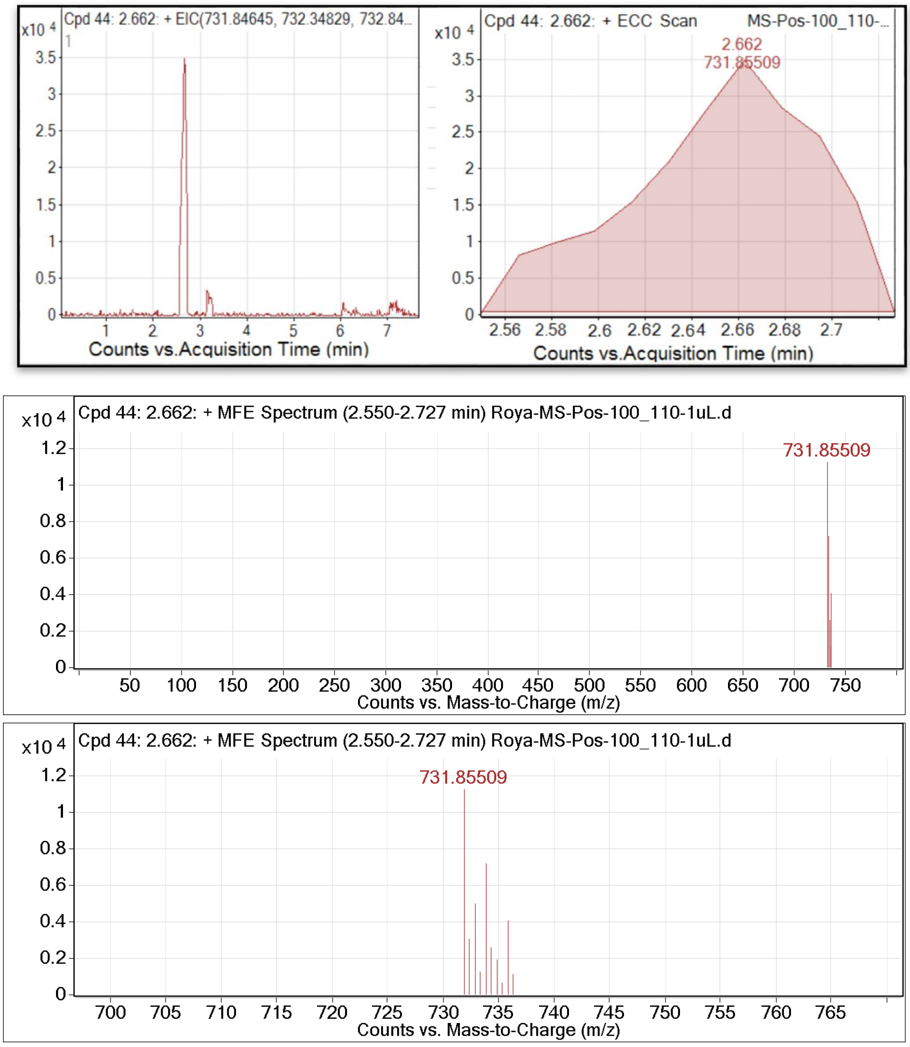

MS Spectrum Peak List

m/z	z	Abundance	Ion
731.85509	2	11223.61	$(M+2H)^{+2}$
732.35531	2	3091.22	$(M+2H)^{+2}$
732.85150	2	4967.72	$(M+2H)^{+2}$
733.35447	2	1258.55	$(M+2H)^{+2}$
733.85727	2	7187.43	$(M+2H)^{+2}$
734.35814	2	2611.45	$(M+2H)^{+2}$
734.84584	2	1935.03	$(M+2H)^{+2}$
735.34231	2	673.28	$(M+2H)^{+2}$
735.85016	2	4069.64	$(M+2H)^{+2}$
736.34987	2	1129.65	$(M+2H)^{+2}$

Supplementary Figure S6. Mass spectroscopy of NTA-DOX. The most abundant ion detected was 731.85509 Da $(M+2H)^{+2}$. The calculated molecular mass by the Chemdraw software was 730.22 Da.

Cellular Uptake of Doxorubicin

OVCAR-3 and 3T3cells (10^5 cells per well) were seeded in six-well plates and incubated for 24 h. Then, free doxorubicin (DOX), HisHBcAg nanoparticles conjugated non-covalently with NTA-DOX (HisHBcAg-NTA-DOX), and HisHBcAg nanoparticles conjugated covalently with folic acid (FA) and non-covalently with NTA-DOX (FA-HisHBcAg-NTA-DOX) were added at equivalent DOX concentration (5 μg/mL). After washing 3 times with ice-cold PBS (pH 7.4), lysis buffer [50 mM Tris (pH 7.4), 0.8% Triton, 0.2% SDS] (0.5 mL per well) was added to lyse the cells. Internalised DOX was measured at A_{490} using a microplate reader (ELX 800; BioTeck Instruments, Winooski, VT, USA) and the results are shown in Supplementary Figure S7.

(**a**)

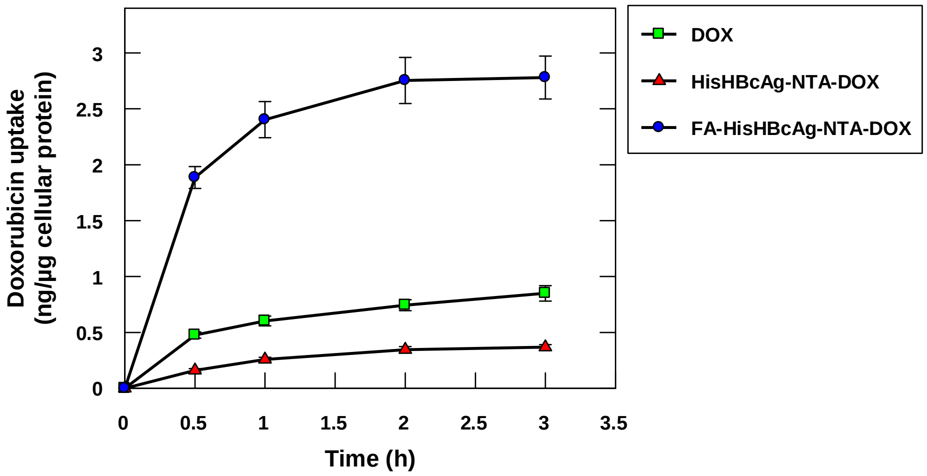

(**b**)

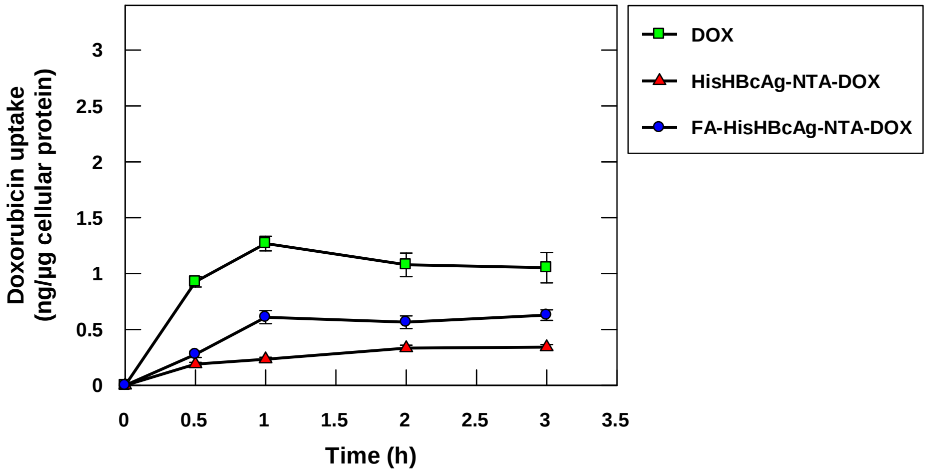

Supplementary Figure S7. Doxorubicin uptake by ovarian cancer and normal cells. Cellular uptake of doxorubicin by (**a**) cancer OVCAR-3, and (**b**) normal 3T3 cells incubated with free doxorubicin (DOX), HisHBcAg nanoparticles conjugated non-covalently with NTA-DOX (HisHBcAg-NTA-DOX), and HisHBcAg nanoparticles conjugated covalently with folic acid (FA) and non-covalently with NTA-DOX (FA-HisHBcAg-NTA-DOX). Data represent mean ± SD of triplicate determinations.
